# Novel Drug Targets in Diastolic Heart Disease

**DOI:** 10.3390/ijms26168055

**Published:** 2025-08-20

**Authors:** Teagan Seng-Mei Er, Boris Martinac, Livia C. Hool

**Affiliations:** 1 School of Human Sciences, The University of Western Australia, Crawley, WA 6009, Australia; teagan.er@research.uwa.edu.au; 2 Victor Chang Cardiac Research Institute, Darlinghurst, NSW 2010, Australia; b.martinac@victorchang.edu.au; 3 St Vincent’s Clinical School, Faculty of Medicine, The University of New South Wales, Darlinghurst, NSW 2010, Australia

**Keywords:** cytoskeleton, diastolic heart disease, mitochondria, L-type calcium channel, PIEZO channels

## Abstract

Diastolic heart failure, also referred to as heart failure with preserved ejection fraction (HFpEF), is a complex cardiovascular clinical syndrome that is a growing health burden worldwide. Patients present with high abnormal left ventricular filling pressures but normal ejection fraction that can progress to diastolic heart failure and death. The causes of diastolic dysfunction are varied, and pharmacotherapies are limited to managing the symptoms of the disease. At the level of the myocyte, cytoskeletal disarray and mitochondrial dysfunction are common features associated with diastolic disease. Understanding the mechanisms of abnormal diastolic filling pressures is necessary to identify novel treatments, which remains an area of significant unmet need. In this article, we discuss the mechanisms of maladaptive feedback contributing to increased extracellular stiffness, cytoskeletal disarray, and mitochondrial dysfunction in diastolic heart failure. Since the mechanisms are complex, understanding the contributing factors provides opportunities for the development of novel drug targets. These will be discussed and examined in the context of current therapy.

## 1. Introduction and Background

Heart failure is a significant global public health issue, and the incidence and prevalence of heart failure are increasing worldwide. Approximately 50% of patients diagnosed with heart failure have a 5-year life expectancy, while those with severe heart failure and NYHA class IV typically die within one year [1]. Additionally, heart failure is the end stage of many disease processes, and the risk of developing heart failure increases with age [2]. Heart failure with reduced ejection fraction (<40%) or systolic failure (also known as HFrEF) is associated with pulmonary congestion, dyspnea, edema, fatigue, and impaired exercise tolerance. It is a progressive disease process, and progression is associated with decreased survival, regardless of underlying etiology.

Approximately half of all heart failure patients develop diastolic dysfunction with preserved ejection fraction (>50%). This is called HFpEF. Several factors conspire to cause HFpEF, in particular the presence of hypertension, renal disease, metabolic syndrome, diabetes, and aging. Additionally, patients with hypertrophic or diabetic cardiomyopathy can progress to HFpEF in advanced stages of disease, suggesting that HFpEF is an advanced form of diastolic heart disease. Despite the varying factors influencing the development of HFpEF, the cardiac phenotype remains relatively consistent, exhibiting concentric left ventricular hypertrophy and diastolic dysfunction (as a result of myocardial stiffening and fibrosis) coupled with systemic inflammation, microvascular endothelial dysfunction, and elevated natriuretic peptides.

It is argued that HFpEF consists of two main mechanisms—hypertensive ventricular hypertrophy and metabolic alterations involving increased oxidative stress coupled with decreased nitric oxide-soluble guanylate cyclase–protein kinase G (PKG) signaling. Hypertension increases mechanical stress through sheer force, activating pro-hypertrophic and pro-fibrotic signaling pathways in the heart, prompting the development of fibrosis and hypertrophy. Metabolic alterations in the heart increase passive stiffness through the phosphorylation of titin by the nitric oxide-soluble guanylate cyclase–PKG signaling pathway [3]. Excessive ROS production during oxidative stress also promotes pro-hypertrophic and pro-fibrotic signaling pathways in the heart. Clinically, HFpEF patients can exhibit either a hypertensive HFpEF phenotype, metabolic HFpEF phenotype, or a combination, suggesting that multiple mechanisms can contribute to HFpEF in some patients. The broad generalization of the HFpEF patient cohort despite varying HFpEF subtypes makes clinical treatment difficult.

Treatments for HFpEF have largely concentrated on managing hypertension and insulin resistance, as well as alleviating disease symptoms. Although the management of comorbidities may alleviate symptoms, there is limited clinical evidence suggesting that these therapeutic strategies improve diastolic function. Pharmacology targeting the sodium glucose transporter 2 (SGLT2) with SGLT2 inhibitors has demonstrated efficacy in reversing HFpEF and is effective in the absence of diabetes. However, SGLT2 inhibitors do not directly target the pathophysiological mechanisms that directly involve the heart. There remains an unmet need for therapeutic strategies that target cardiac-related mechanisms in HFpEF.

**Figure 1:**
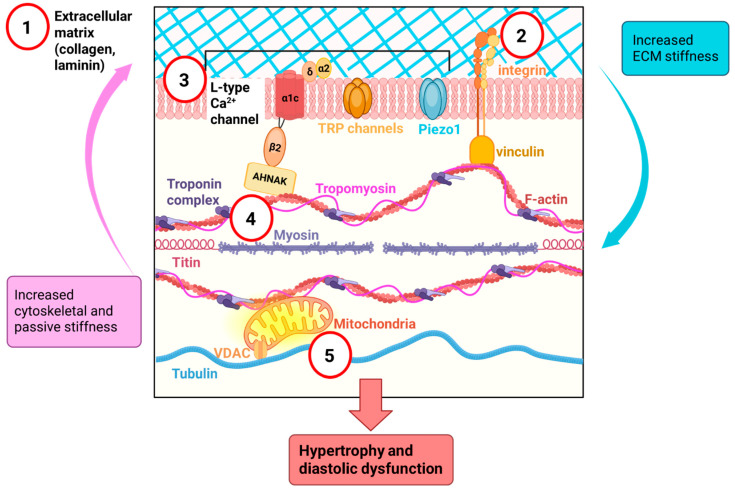
A schematic of the maladaptive feedback loop which contributes to hypertrophy and diastolic dysfunction. The extracellular matrix (1), the costamere (complex of proteins involving integrin and vinculin) (2), ion channels (3), the sarcomere and cytoskeleton (4), and mitochondria (5) are all involved in the maladaptive feedback mechanism, which is involved in the development and the persistence of cardiac hypertrophy and diastolic dysfunction. Abbreviations: AHNAK = neuroblast-associated differentiation protein/desmoyokin, TRP = transient receptor potential, VDAC = voltage-dependent anion channel.

The complex mechanisms involved in HFpEF pathophysiology contribute significantly to the lack of translatability of therapeutics into the clinic. The progression of fibrosis, increased extracellular matrix (ECM) and myocardial stiffness, cytoskeletal disarray, altered cellular and nuclear signaling, and altered mitochondrial energetics are closely linked but are often targeted in isolation. Alterations in signaling, mitochondrial function, and the cytoskeleton also occur early before the development of hypertrophy or fibrosis. Furthermore, the recapitulation of HFpEF in murine models developed as a result of metabolic and hypertensive stress [4,5,6] or nitrosative stress [7] has progressed preclinical investigations into HFpEF but still lacks the mechanistic complexity seen in the human condition. To improve the efficacy of therapeutics for HFpEF, the mechanisms involved in HFpEF must be better understood. In this article, we examine the mechanisms for the contributing factors and discuss therapeutics that could target these sites to ameliorate the maladaptive feedback and prevent the development of HFpEF. We also discuss the translational considerations which must be taken into account when transitioning the preclinical mechanistic findings into therapeutics for the clinic. We have identified five sites as potential targets for therapy (Figure 1) and discuss how each site contributes to diastolic disease and propose therapies to prevent progression of disease.

## 2. Drug Target: The Extracellular Matrix

The cardiac extracellular environment is composed of a network of collagens, proteoglycans, and glycoproteins including fibronectins, elastin, and laminins [8]. These proteins contribute to cell adhesion, cell motility, and mechanical cue communication intracellularly to regulate intracellular stiffness by altering the cytoskeleton. The ECM undergoes constant remodeling under the influence of inflammatory, chemical, and mechanical cues to maintain homeostasis. Cardiac remodeling can either be physiological (e.g., in athletes and pregnancy) or pathological (e.g., HFpEF). Myocardial stiffening is a well-recognized consequence of dysregulated cardiac remodeling, resulting in reduced left ventricular compliance, impaired electrical conductance, and diastolic dysfunction.

Fibrosis contributes to diastolic heart disease and HFpEF. In fact, HFpEF patients exhibit a pro-fibrotic profile with elevated plasma levels of fibroblast growth factor-23, YKL4O, ST2, and metalloprotease (MMP)-2 [9]. Myocardial biopsies from HFpEF patients exhibit an accumulation of collagen in the interstitial space and a reduction in MMP-1 expression [10]. These alterations in protein expression and deposition contribute to increased fibrosis and subsequent myocardial stiffening and diastolic dysfunction.

Hydrogel technology has enabled the recapitulation of varying ECM stiffnesses as in vitro models of disease to examine the impact of ECM stiffness on cardiac myocyte function. H9c2 myoblasts or wildtype mouse ventricular cardiac myocytes that are plated on stiff hydrogels develop alterations in cell signaling and protein expression that mimic the hypertrophic heart [11]. Specifically, plating wildtype cardiac myocytes on stiff hydrogels increased integrin and vinculin expression and recapitulated the hypermetabolic state evident in hypertrophic cardiomyopathy [11]. It is proposed that this leads to a maladaptive feedback loop between the ECM and the cytoskeleton, which also involves ion channels, integrin, and the mitochondria (Figure 1). The maladaptive feedback loop drives the development and persistence of cardiac myocyte hypertrophy and diastolic dysfunction. By blocking integrin, a key protein in transducing signals between the ECM and the cytoskeleton, with an antibody directed against integrin, we were able to abolish the hypermetabolic state. Conversely, cardiac myocytes derived from hypertrophic cardiomyopathic mice (carrying a mutation in troponin I (cTnI-G203S)) plated on soft hydrogels displayed a reduction in integrin and vinculin expression. These findings emphasize the importance of the ECM, cytoskeleton, and mitochondrial crosstalk in the development of diastolic dysfunction. Since ECM stiffness may play a crucial role in the maladaptive feedback loop perpetuating diastolic dysfunction, identification of therapeutics that target the ECM to reduce myocardial stiffness is a promising therapeutic avenue. In this section, we will describe the different aspects of the ECM that influence myocardial stiffness and diastolic dysfunction, as well as potential therapeutic targets.

### 2.1. Regulation of ECM Protein Synthesis

An increase in ECM protein synthesis is well documented in HFpEF, as well as in diabetic, hypertensive, and hypertrophic cardiomyopathy [12,13,14]. Collagen type I and collagen type III fibers predominate in the heart, contributing to 85% and 11% of collagen fibers in the myocardium [15]. In diastolic heart disease, the expression of collagen types is altered, resulting in a change in the ratio of collagen type I to collagen type III, contributing to fibrosis. Alterations in ECM protein gene expression or MMP expression contribute to the alterations in ECM protein synthesis evident in HFpEF.

The rate of collagen turnover is used as an indirect measure of collagen gene expression in the heart. The synthesis of collagen type I and collagen type III is measured with the serum markers carboxy-terminal propeptide of type I procollagen (PICP) and amino-terminal propeptide of type I and III procollagen (PINP and PIIINP, respectively) [16]. Degradation of collagen I can also be measured using the serum marker carboxy-terminal telopeptide of collagen type I (ICTP) [16]. Alterations in the serum levels of PICP, PIIINP, and ICTP are evaluated to determine alterations in collagen turnover in patients compared to healthy controls. An increase in synthesis markers coupled with a reduction in collagen I degradation is associated with fibrosis and is commonly reported in heart failure patients [17,18,19]. The excessive deposition of collagens and other ECM proteins in the absence of sufficient degradation processes in heart failure is maladaptive and contributes to the development of diastolic dysfunction. The increased collagen deposition perpetuates the maladaptive signaling between the ECM and the cardiac myocytes, promoting cardiac myocyte hypertrophy and fibrosis. Ultimately, the fibrosis from the excessive deposition of ECM proteins results in an increase in passive stiffness coupled with increased ventricular filling pressures, promoting the development and persistence of diastolic dysfunction.

The increase in ECM protein expression in diastolic heart disease is coupled with changes in the expression of MMPs and tissue inhibitors of metalloproteases (TIMPs). MMPs and TIMPs control the degradation of ECM proteins in the extracellular matrix, providing an alternative mechanism to regulate ECM protein synthesis that does not involve altering ECM protein gene expression. The balance of MMP and TIMP expression is finely regulated to control ECM protein turnover. It has been well documented that in hypertensive and hypertrophic cardiomyopathy patients, there is a reduction in circulating and tissue levels of MMP-1 and an increase in TIMP-1, MMP-2, and MMP-9 [14,20,21,22]. Furthermore, complete inhibition of MMP-9 has been associated with the attenuation of left ventricular remodeling and collagen accumulation in a model of pressure overload hypertrophy, whilst MMP-9 activation exacerbates cardiac dysfunction [23,24]. MMP expression is commonly assessed in conjunction with collagen synthesis and degradation serum markers to establish a pro-fibrotic phenotype in HFpEF patients.

### 2.2. ECM-Mediated Signaling Pathways

ECM protein synthesis and the MMP/TIMP profile can be regulated by the tissue growth factor β (TGF-β)/SMAD signaling pathway. TGF-β is expressed in cardiac myocytes, fibroblasts, and the ECM. The TGF-β signaling cascade promotes cardiomyocyte growth, stimulates fibroblast proliferation, and enhances ECM protein synthesis whilst simultaneously suppressing proteins that degrade the ECM [25,26]. In fact, elevated TGF-β levels have been reported in hypertrophic and diabetic myocardium and correlate with increased fibrosis in pressure overload hypertrophy [25,27].

TGF-β is documented to promote ECM protein synthesis through TGF-β’s regulation of collagen expression through collagen promoter genes [15,28]. This regulation occurs in cardiac fibroblasts, promoting fibroblast differentiation into myofibroblasts and production of collagen type I and III. Subsequently, myofibroblasts are activated and begin to remodel the ECM. When unregulated, ECM remodeling by fibroblasts becomes maladaptive, and the myocardium stiffens, resulting in diastolic dysfunction. TGF-β signaling has also been reported to be influenced by integrins, plasma membrane-embedded proteins which interact with ECM proteins, to communicate between the ECM and the cytoskeleton. Activation of latent TGF-β by integrins can promote fibrosis by stimulating fibroblast differentiation and the activation of pro-hypertrophic and pro-fibrotic signaling cascades in cardiac myocytes. Hence, TGF-β acts as an important signaling molecule in the maladaptive feedback loop between the ECM and the heart as TGF-β can enhance pathological ECM remodeling.

### 2.3. Therapeutics Targeting the ECM and ECM-Related Signaling Pathways

As the understanding of how ECM remodeling contributes to myocardial stiffening and diastolic dysfunction evolves, new therapeutic strategies have been developed and tested. Therapeutics can target the regulation of protein synthesis through inhibition of MMPs, target ECM signaling pathways, or target comorbidities, which contribute to the development of fibrosis.

Owing to the importance of MMPs in ECM degradation, strategies have been developed to target MMPs to reduce fibrosis in the heart. However, due to the vast number of MMPs, selective inhibition of MMP subtypes results in a compensatory increase in the expression of other MMPs, thereby limiting the efficacy of MMP treatment in cardiac fibrosis reduction [26]. Furthermore, some MMP therapies have been reported to cause deleterious effects such as arthralgia and/or myalgia in clinical trials [29,30]. Therefore, other strategies have been sought to target ECM protein synthesis.

ECM signaling pathways are a promising therapeutic target as they regulate ECM protein synthesis. So far, direct inhibition of TGF-β has shown promise in preclinical murine models with the reduction in fibrosis coupled with an improvement in diastolic function [31]. However, TGF-β inhibitors such as fresolimumab have not successfully progressed in human trials due to adverse events [32]. As TGF-β has anti-proliferative and immunosuppressant effects, TGF-β inhibition may have carcinogenic effects and can contribute to the development of autoimmunity and inflammation in patients. Pirfenidone is a US Food and Drug Administration-approved TGF-β inhibitor for idiopathic pulmonary fibrosis that has shown promise in targeting cardiac fibrosis in preclinical animal models. However, the drug has been reported to have deleterious gastrointestinal and skin-related adverse effects [33]. If TGF-β inhibition were to be pursued to reduce fibrosis in the heart, studies would need to investigate ways to target cardiac-specific TGF-β or cardiac-specific upstream or downstream targets to limit adverse effects on other organs.

A therapeutic target upstream from TGF-β that is used clinically to treat cardiac diseases is the angiotensin II receptor. Antagonism of the angiotensin II receptor is a therapeutic strategy currently used in the treatment of hypertrophic cardiomyopathy and hypertensive hypertrophy [34]. A clinical trial demonstrated that losartan, an angiotensin II receptor antagonist, was able to attenuate fibrosis progression in both hypertrophic cardiomyopathy and hypertensive cardiomyopathic patients [26,35,36]. Another clinical study investigating the angiotensin-converting enzyme inhibitor lisinopril also demonstrated a reduction in fibrosis in hypertensive patients [26,37]. Although angiotensin II inhibition has shown beneficial effects on fibrosis in the heart, angiotensin II inhibition has limited efficacy in patients with HFpEF, as there is no improvement in diastolic function [26,38,39,40]. The etiology of HFpEF is diverse and can include hypertension, diabetes, and metabolic syndrome. The use of losartan and lisinopril relieves the hypertension by ameliorating the vascular pathophysiology present and reduces fibrosis caused by hypertensive stimuli. However, losartan and lisinopril do not directly improve other cardiac-related mechanisms contributing to the HFpEF phenotype. As angiotensin II receptor antagonism has limited efficacy in HFpEF, the anti-fibrotic effects of these drugs are not sufficient to disrupt the maladaptive feedback mechanism between the ECM and cardiac myocytes contributing to the diastolic dysfunction.

SGLT2 inhibitors are prescribed to patients with diabetes and more recently in heart failure patients [34]. Although SGLT2 inhibitors do not directly target the ECM or fibrosis, it has been reported that the inhibitors can reduce pathological ECM remodeling in the heart [41,42,43,44,45]. Zhu et al. proposed that the positive effect of the SGLT2 inhibitor dapagliflozin involves the angiotensin I receptor/phosphorylated focal adhesion kinase (p-FAK)/NADPH oxidase (NOX) 2 pathway [41]. Angiotensin I receptor and p-FAK are proteins involved in mechanotransduction, and NOX2 is a major source of reactive oxygen species (ROS). The expression of these proteins increases as diabetic cardiomyopathy disease progresses. The proteins also increase when H9c2 myoblasts are plated on stiff hydrogels, suggesting that protein expression correlates with ECM stiffening during disease progression [41]. Treatment with dapagliflozin can attenuate the increase in collagen deposition associated with disease progression and can also reduce the expression of angiotensin I receptor, p-FAK, and NOX2 [41]. These findings support the relationship between myocardial stiffness and disease progression in diabetic cardiomyopathy. Interestingly, dapagliflozin and empagliflozin’s efficacy is negatively associated with increased myocardial stiffness [41,46], suggesting that early treatment would be more effective in the reduction in fibrosis.

Notably, the different therapeutic strategies all target aspects of the maladaptive feedback loop. Therapeutic strategies targeting the ECM using MMP or TGF-β1 inhibitors, as well as SGLT2 inhibitors, can also affect integrin signaling by either acting as a substrate for integrin (MMPs and TGF-β1; expanded on in Section 3) or by influencing integrin adaptor proteins (dapaglifozin: p-FAK). These therapeutic mechanisms highlight the close association between the ECM and integrin signaling. Therefore, designing anti-fibrotic therapies may be more effective when targeting specific mechanisms that involve the maladaptive feedback loop, especially the interactions between the ECM and integrin, which will be described further in Section 3.

### 2.4. Clinical Evidence

Aberrant ECM remodeling and subsequent myocardial stiffening in diastolic heart disease contribute significantly to the clinical presentation of patients with HFpEF and in aged hearts. Fibrosis contributes to the presentation of dyspnea and exercise intolerance in patients with HFpEF and impacts the patient’s quality of life. In some cases, fibrosis precedes the development of diastolic dysfunction. Therefore, targeting ECM remodeling to relax the myocardium is an attractive therapeutic strategy to improve symptoms and quality of life in patients. Indeed, many clinical trials have reported reductions in fibrosis with angiotensin II receptor antagonists and SGLT2 inhibitors in patients with cardiac pathologies [45,47]. However, whether the reduction in fibrosis is coupled with improvements in cardiac functional parameters and patient quality of life differs between clinical trials. Clinical trials directly targeting the mechanisms which promote fibrosis or degrade ECM proteins such as MMPs and TGF-β also fail to progress through clinical trials due to adverse off-target effects, limiting the application of current ECM-targeted therapeutics in the clinic.

### 2.5. Translational Considerations

Although the development of therapeutic strategies which target aberrant ECM remodeling has shown promise in preclinical models, these strategies often fail to progress through clinical trials. Several barriers exist in the translation of these therapeutic strategies to the clinic, including the non-specific action of therapies, the complex interplay between the ECM and the cellular environment, and the diverse etiology of HFpEF.

A prominent barrier in the translation of therapeutics targeting the ECM in HFpEF is the non-specific action of MMP and TGF-β inhibition. Fibrosis is not unique to the heart; it is also evident in the lungs, liver, kidneys, and vasculature. Consequently, the mechanisms that regulate the composition of the ECM are not cardiac-specific despite the strong correlation between collagen serum markers and cardiac fibrosis. Clinical trials inhibiting either MMPs or TGF-β have reported deleterious off-target effects and are thus not suitable for treating cardiac fibrosis. Therefore, there is a need for novel therapeutic strategies that reduce cardiac fibrosis in the absence of off-target effects. Such therapeutic strategies should target cardiac-specific pathways that regulate fibrosis in the heart.

Recently, SGLT2 inhibitors have shown promise in reducing cardiac fibrosis and improving diastolic function in HFpEF in the absence of deleterious adverse events. However, the efficacy of SGLT2 inhibitors has been reported to be dependent on the extent of cardiac fibrosis [41,46], limiting the applicability of SGLT2 inhibitors in reducing cardiac fibrosis. Developing a therapeutic strategy that uses a cocktail therapy approach combining SGLT2 inhibitors with other anti-fibrotic therapies should be investigated to improve the efficacy of SGLT2 inhibitors when fibrosis is well-established. Additionally, understanding the mechanistic pathways that contribute to the maladaptive feedback loop between the ECM and the cardiac myocytes may provide further insight into potential therapeutic targets to reduce fibrosis.

The diverse etiology of HFpEF also acts as a translational barrier. HFpEF is a term that broadly defines patients with heart failure in the presence of preserved ejection fraction. The patient demographic for HFpEF is diverse, with some patients exhibiting a more metabolic or hypertensive phenotype. Furthermore, both diabetic and hypertrophic cardiomyopathy can progress into HFpEF in advanced stages of the disease. As the etiology for patients with HFpEF is diverse, therapeutic strategies that are effective in some patients may not be effective for other patients. In the context of ECM-targeted therapeutics, these strategies may be more effective in hypertensive HFpEF patients, as fibrosis is a major consequence of hypertension. Therefore, more personalized therapeutic strategies need to be developed to treat different subsets of HFpEF patients to improve patient outcomes.

## 3. Drug Targets: Costamere

**Figure 2:**
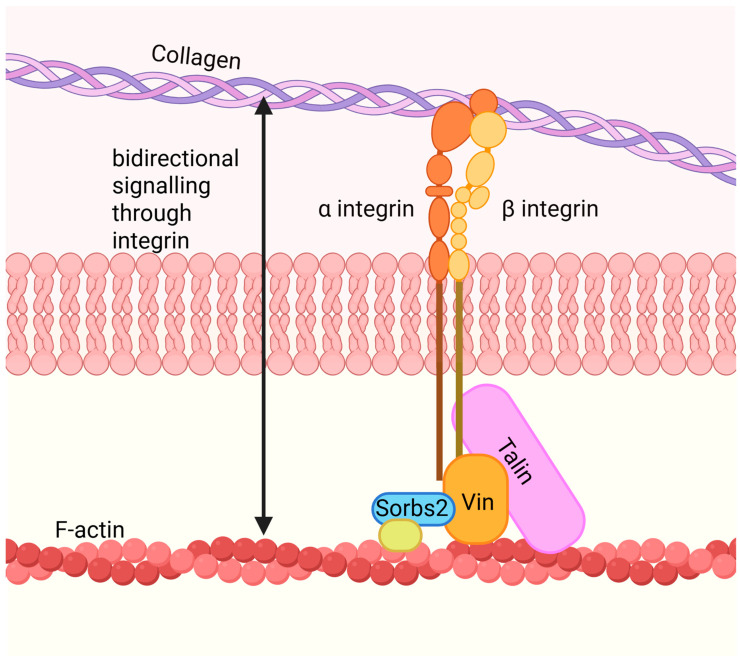
The costamere. The costamere is a complex of proteins that form focal adhesion points that are involved in the mechanotransducive properties of cardiac myocytes. Integrin is a transmembrane protein embedded into the plasma membrane that transduces signals bidirectionally between the extracellular matrix and the cytoskeleton as indicated by the arrow. The mechanosensitive properties of integrin are assisted by the presence of adaptor proteins such as talin and vinculin. Adaptor proteins such as SORBS2 also participate in the mechanotransduction of signals across the costamere by interacting with vinculin and the cytoskeleton. Abbreviations: Vin = vinculin. SORBS2 = sorbin and SH3 domain containing 2.

The costamere is a complex of proteins involving integrin and its adaptor proteins (e.g., vinculin, talin-1, and talin-2) (Figure 2). The main role of the costamere is to transmit signals between the ECM and the sarcomere and vice versa. Upon an increase in mechanical load, focal adhesions at the site of the costamere increase, enhancing the structural–functional communication between the ECM and the sarcomere. This is important as ~20–30% of force generated by striated muscle is lateral [48,49]. Owing to the costamere’s crucial role in communicating between the ECM and the sarcomere, it acts as a possible therapeutic target to disrupt the maladaptive feedback loop that contributes to the development and persistence of diastolic heart disease. In this section, we will describe the different elements of the costamere and therapies targeting these proteins to treat diastolic heart disease.

### 3.1. Integrin

Integrin is a structural transmembrane protein that is capable of transducing mechanical forces across the plasma membrane between the ECM and intracellular environment. Integrins form heterodimers, and cardiac myocytes exclusively express the heterodimers α1β1, α5β1, and α7β1 [50]. These heterodimers act as receptors for collagen, fibronectin, and laminin, respectively [50]. Integrins can transduce signals bidirectionally with the assistance of adaptor proteins. Outside-in signaling involves signals being transmitted from the ECM into the intracellular environment to alter cytoskeletal arrangement and activate signaling pathways [50]. Inside-out signaling involves signals from the intracellular environment that then alter the ECM by modulating integrin affinity [50]. Importantly, integrin interacts with the actin cytoskeleton to regulate actin rearrangement and to transmit signals to the nucleus [51]. As the sensing of mechanical stress and myocardial stiffness is considered a main driver triggering the development and persistence of disease, integrin could be targeted to treat HFpEF.

#### 3.1.1. Integrins in Diastolic Heart Disease

The role of integrin in diastolic heart disease has been well described in the literature in preclinical animal models [11,41,52]. An upregulation of cardiac-specific β1 integrin has been measured in diabetic cardiomyopathic rats and in a mouse model of hypertrophic cardiomyopathy, highlighting its role in disease [11,41]. Integrin expression is proposed to be upregulated in response to the stiffening of the myocardium and the cytoskeleton. Consistent with this argument, plating rat or mouse ventricular myocytes on stiff hydrogels increased integrin β1 expression, whereas plating hypertrophic mouse ventricular myocytes on soft hydrogels (that mimic the healthy myocardium) reduced integrin β1 expression [11]. Application of an integrin-blocking antibody attenuated the hypermetabolic phenotype in wildtype cells plated on stiff hydrogels [11], emphasizing the importance of integrin in the communication between the ECM, cytoskeleton, and mitochondria in cell physiology.

The mechanosensitive role of integrin can be confirmed by alterations in integrin β1 expression across different hydrogel stiffnesses. The transmission of mechanosensitive signals through integrin activates focal adhesion kinases and other downstream signaling pathways that play a key role in ECM remodeling and hypertrophy. Mechanical stress from comorbidities such as hypertension can contribute to the activation of pro-hypertrophic and -fibrotic signaling pathways via integrin. The ability for integrin to transmit signaling from mechanical stress highlights the potential of integrin-based therapeutics to interrupt the maladaptive signaling between the ECM and the cytoskeleton to prevent and reverse hypertrophy and diastolic dysfunction.

Integrin function can also be altered in diastolic heart disease through alterations in the proteins/growth factors integrin interacts with. It is well established that diastolic heart disease is coupled with increased ECM protein deposition and altered TGF-β and MMP expression. Integrin α_5_β_1_ has been reported to bind latent TGF-β1, and MMPs have been documented to interact with integrin heterodimers [53]. As integrins can potentiate signals from the ECM, TGF-β, and MMPs [54], the altered expression patterns in cardiac pathology can alter the signaling between the ECM and the cytoskeleton through integrin. An increase in TGF-β expression coupled with the increase in β1 integrin in diastolic heart disease can increase pro-fibrotic signaling in the heart and contribute to the maladaptive ECM remodeling.

The upregulation of α integrins such as α11 has also been linked to diabetic cardiomyopathy through TGF-β2. Knockdown of α11 in diabetic streptozotocin-treated Sprague-Dawley rats attenuated the increase in TGF-β2 transcription in cardiac fibroblasts [55]. TGF-β2 transcription is proposed to mediate myofibroblast differentiation into cardiac fibroblasts; therefore, a reduction in TGF-β2 transcription attenuates the increase in fibrotic deposition [55]. The combined increase in interactions among integrins, ECM proteins, TGF-β, and MMPs in cardiac pathology can contribute to enhanced integrin-derived signaling between the ECM and the cardiac myocytes, perpetuating the maladaptive feedback loop, contributing to diastolic dysfunction.

#### 3.1.2. Therapeutic Strategies to Target Integrins

Integrin function can be modulated with the application of small-molecule inhibitors or monoclonal antibodies. Small molecules are a more attractive delivery agent as they are cost-effective, can be orally administered, and are generally safer than monoclonal antibodies [8]. However, complete inhibition of integrin subtype function is not an effective therapeutic strategy. Integrin expression of α and β subtypes is not organ-specific, and although α1, α5, and α7 are expressed in cardiac tissue, other integrin subtypes are also expressed in cardiac pathology. It is important to note that evidence for the use of integrin inhibitors in the context of treatment of cardiomyopathy is limited. Cardiovascular clinical trials have focused on inhibiting integrin function as an antithrombotic agent to treat acute coronary syndrome but have not tested integrin inhibition in patients with HFpEF [8].

As complete integrin inhibition is potentially maladaptive, therapeutic strategies have been developed that target specific integrin functions. As fibrosis is associated with increased TGF-β, therapeutics have been developed that target the TGF-β and integrin interaction. αvβ1, αvβ5, αvβ6, αvβ8, and α5β1 integrins can activate latent TGF-β and potentiate signaling derived from TGF-β [54]. By activating latent TGF-β, integrins can regulate ECM remodeling by controlling active and latent TGF-β expression. PLN-74809, an integrin αvβ6 and integrin αvβ1 inhibitor, has successfully progressed through phase I (healthy individuals) and phase II clinical trials for idiopathic pulmonary fibrosis and displays a good safety profile [56]. However, despite the promising outcomes from therapeutics targeting the TGF-β-integrin interaction, evidence for cardiac-specific therapeutic approaches remains limited.

Therapeutic strategies which target downstream effectors of integrin have also been investigated to disrupt the maladaptive signaling between the ECM and cytoskeleton. As integrin lacks enzymatic activity, it relies on adaptor proteins to activate downstream signaling pathways (covered more extensively in Section 3.2) and to alter integrin binding affinity to ECM proteins. By targeting integrin’s interactions with adaptor proteins, integrin function does not need to be completely inhibited, avoiding potential adverse events that arise from complete integrin inhibition.

#### 3.1.3. Clinical Evidence

Despite the importance of clinical evidence to translate integrin-targeted therapeutic strategies into the clinic, the evidence remains limited in HFpEF and diastolic heart disease. To date, the majority of integrin inhibitors that have been tested in the context of cardiovascular disease are used to treat acute coronary syndrome and thrombotic cardiovascular events. These include batifiban (CTR20130814), pury peptide (CTR20170691 and CTR20181547), and HYC-11395 (CTR20182266) [54]. Although these integrin inhibitors are used for acute coronary syndrome, they do not target the heart. Rather, these integrin inhibitors act as antithrombotic agents as they target αIIbβ3 on platelets [54]. There is also a lack of evidence for integrin expression in human cardiac samples in both healthy individuals and in the presence of cardiac pathology. As integrins play a crucial role in the maladaptive feedback, contributing to diastolic dysfunction, studies should investigate the clinical relevance of integrin in diastolic heart disease and develop therapeutic targets that could disrupt integrin’s ability to communicate the maladaptive signaling between the ECM and the cytoskeleton.

#### 3.1.4. Translational Considerations

A prominent barrier in the translation of integrin-based therapeutics for the treatment of HFpEF is the broad expression of integrins throughout the body. As integrin heterodimers consist of a combination of α and β subunits, therapeutic strategies should be optimized to target cardiac-specific combinations of α and β integrins to limit off-target effects.

β1 integrin would appear to be an ideal therapeutic target due to its involvement in myocardial stiffness and cardiomyopathy in preclinical models and its exclusive expression as the main β integrin subtype in the cardiac myocyte. However, β1 is not exclusively expressed in the heart. It is also expressed in immune cells [57,58], the kidneys [59], the pancreas [60], and the colon [61]. As β_1_ is expressed across various organs, small-molecular inhibitors or monoclonal antibodies have the potential to target the heart, immune cells, the kidneys, the pancreas, and the colon, resulting in the potential for off-target effects. Similarly, the combination of α1β1, α5β1, and α7β1 is expressed in other tissue types. For instance, α7 or α7β1 deficiency is associated with muscular dystrophy or abnormal cerebral vascular smooth muscle cell recruitment and survival [62,63]. As targeting integrin could result in deleterious off-target effects, this must be considered when translating integrin-based therapies into the clinic.

Another barrier in translating integrin-targeted therapeutic strategies is the presence of compensatory mechanisms. The integrin heterodimer can consist of different combinations of α and β integrin subunits. In cardiac myocytes, β1 integrin is the only β subunit expressed, but there are three different α integrin subunits (α1, α5, and α7) that can dimerize with β1. An upregulation of integrins such as α11 has also been documented in stress- and pressure-overloaded hearts [64]. Therefore, preferential inhibition may result in a compensatory increase in another. Proteins other than integrin can also be upregulated to compensate for the loss of integrin. A double knockdown of α11 integrin and syndecan-4 was demonstrated to prevent cardiac hypertrophy in aortic-banded rats, whereas knockdown of α11 integrin alone was unable to prevent cardiac hypertrophy [64]. Hence, the efficacy of small molecules and monoclonal antibodies that target integrin may be reduced due to compensatory mechanisms in the heart.

As integrin inhibition has the potential for off-target effects and reduced efficacy through compensatory mechanisms, the translation of integrin inhibitors into the clinic is difficult for HFpEF. The translation of integrin-based therapies into the clinic requires pharmacological agents that can specifically target cardiac integrins without completely inhibiting integrin function. Targeting integrin adaptor protein function and/or interactions with integrin that are cardiac-specific and contribute to maladaptive responses may be more effective as a therapeutic target to treat HFpEF and will be discussed in the following section.

### 3.2. Adaptor Proteins

Although integrin is an essential protein for transmitting signals between the ECM and intracellular environment, integrin lacks enzymatic activity. Adaptor proteins such as talin-1, talin-2, vinculin, and sorbin and SH3 domain containing 2 (SORBS2) are coupled to integrin or other adaptor proteins to transduce signals between the ECM and intracellular environment. Importantly, talin and vinculin functionally couple integrin to the actin cytoskeleton in the adult heart, highlighting their importance in linking the ECM, integrin, and the cytoskeleton in the maladaptive feedback model [8,65].

Talin-1 and talin-2 are important integrin adaptor proteins that transduce signals between the actin cytoskeleton and ECM through integrin. The activation of integrins and the formation of focal adhesion points/costameres are dependent on the binding of talin to the plasma membrane [66]. Talin-1 and talin-2 are both expressed in the heart during embryogenesis, with talin-1 being the predominant isoform. In the adult heart, the expression pattern shifts to talin-2 as the predominantly expressed isoform [67]. Interestingly, in a model of pressure overload-induced cardiac hypertrophy, talin-1 expression was upregulated at the costamere [67]. The exclusive expression of talin-1 at the costamere suggests that talin-1 plays an important role in the communication between the sarcomere and the ECM during cardiac stress and pathology. Knockdown of talin-1 in mice was able to blunt pressure overload-induced hypertrophy, reduce fibrosis, and preserve both diastolic and systolic function [67]. These studies demonstrate that talin-1 could be a potential therapeutic target, as it is only implicated in situations of mechanical stress/pathology in the adult heart and should be investigated in the context of diastolic heart disease.

Vinculin is another adaptor protein that interacts with integrin, talin-1/2, and the actin cytoskeleton. Vinculin plays a critical role in cardiac development, tissue remodeling, and cell migration [68,69]. Through vinculin’s interactions with integrin, talin, and actin, vinculin can transmit mechanical force through the cytoskeletal network, promoting actin dimerization and actin filament bundling [70,71].

The role of vinculin in diastolic heart disease remains limited. Owing to the mechanosensitive role of the costamere, vinculin expression is increased in ventricular cardiac myocytes plated on stiff hydrogels [11]. Ventricular myocytes isolated from hypertrophic mice also demonstrate an upregulation of vinculin expression [11]. The relationship between integrins and vinculin in responding to mechanical stress and ECM stiffness indicates that this complex is a crucial element of the maladaptive feedback loop, which contributes to hypertrophy and diastolic dysfunction.

Recently, another adaptor protein, SORBS2, has been identified to be involved in the interactions between integrin, the actin cytoskeleton, and pathological remodeling of the myocardium. SORBS2 is important in cell adhesion, cytoskeletal organization, and growth hormone signaling [72]. Unlike talin and vinculin, SORBS2 interacts with proteins important for the connection between integrins and the actin cytoskeleton rather than directly interacting with integrin [73]. One of the proteins that SORBS2 interacts with is vinculin, emphasizing its biological role in communicating between the sarcomere and the ECM.

Embryonic homozygote loss of SORBS2 (SORBS2^−/−^) in mice can induce a phenotype that resembles dilated cardiomyopathy, congenital heart disease, and arrhythmogenic right ventricular cardiomyopathies. Conversely, an increase in SORBS2 expression is evident in cardiac pathology and stress [73]. In heart failure patient myectomy samples and transaortic constricted mouse hearts, the increased SORBS2 expression is correlated with reduced cardiac function (decreased ejection fraction), hypertrophy (increased left ventricular mass), and increased fibrosis (collagen fractional volume). These results emphasize SORBS2′s role in pathological remodeling and hypertrophy in the failing heart. To investigate the role of SORBS2 in pathological remodeling in adult mice, a tamoxifen-inducible homozygous knockdown of an SORBS2 mouse model was generated. With this model, loss of SORBS2 exacerbated pathological remodeling of the heart, reduced left ventricular volume and cardiac stroke volume, and increased posterior wall thickness in transaortic-banded mice [73]. It is proposed that SORBS2 interactions between integrin, adaptor proteins, and the cytoskeleton influence signaling between the ECM and the cytoskeleton. Hence, SORBS2 may be involved in the maladaptive feedback response.

### 3.3. Therapeutic Strategies Targeting Integrin Adaptor Proteins

Therapeutic strategies targeting integrin adaptor proteins are a novel and attractive therapeutic strategy to treat diastolic heart disease. Preclinical studies have identified that the expression of talin-1 and SORBS2 during mechanical stress and pathology could act as a potential therapeutic target. However, how talin-1 and SORBS2 can be pharmacologically targeted to mitigate the pro-hypertrophic and pro-fibrotic signaling occurring through the costamere remains unexplored.

Reports investigating vinculin as a therapeutic target are limited, so it is unknown whether targeting vinculin could be therapeutically beneficial. As vinculin expression is upregulated in hypertrophic cardiomyopathy, and when plated on stiff substrates, the interaction between vinculin and integrin acts as a novel therapeutic strategy. However, it has been reported that cardiac-specific loss-of-function mutations in vinculin result in dilated cardiomyopathies and sudden cardiac death [74]. As vinculin loss of function is associated with dilated cardiomyopathy, complete inhibition of vinculin function may be maladaptive. Rather, further work should investigate how to alter vinculin’s function/interactions with integrin and actin without completely inhibiting vinculin function to disrupt the maladaptive feedback between the ECM and the cardiac myocyte. Such investigations could provide evidence for vinculin as a therapeutic target.

### 3.4. Clinical Evidence

Clinical evidence supporting a role for integrin adaptor proteins in the clinic remains limited. The majority of the studies have been explored in preclinical animal models, and further work should be performed to characterize these findings either in human-inducible pluripotent stem cells differentiated into cardiac myocytes or in patients.

### 3.5. Translational Considerations

The main barriers to translating therapeutics targeting integrin adaptor proteins are the reported use of preclinical animal models that exhibit both diastolic and systolic heart failure and a lack of therapeutically driven evidence. Studies investigating the role of talin-1/-2 and SORBS2 in the heart are currently restricted to models of pressure overload where both diastolic and systolic dysfunction are present. Considering that HFrEF therapeutics have limited efficacy in HFpEF, preclinical studies and clinical studies investigating the importance of integrin adaptor proteins in diastolic heart disease, where systolic function is preserved, are of clinical importance. Additionally, these preclinical studies have predominantly been mechanistic, using genetic knockout mouse models to assess the functional importance of integrin adaptor proteins in heart failure. Studies have yet to explore how talin-1 and SORBS2 can be pharmacologically targeted to interrupt the maladaptive signaling transmitted through the costamere.

## 4. Drug Targets: Ion Channels

Ion channels play an essential role in the excitability of the cardiac myocyte, as they allow the transport of ions across the plasma membrane. Channelopathies, whereby the channel has altered expression or altered function, are commonly associated with cardiomyopathies and form a crucial part of the maladaptive feedback response in diastolic heart disease. In this section, we will discuss two types of ion channels. First, the L-type calcium channel is essential for cardiac excitation and contraction but also elicits characteristic mechanosensitive responses. Secondly, we will review the therapeutic potential of mechanosensitive channels such as PIEZO and transient receptor potential melastatin 4 (TRPM4) channels, which are activated by mechanical stimuli.

### 4.1. L-Type Calcium Channel

The L-type calcium channel is a voltage-sensitive channel that initiates contraction. The channel consists of the α1c subunit and auxiliary α2, δ, and β subunits [75]. The α1c subunit is voltage-sensitive, and when the plasma membrane potential reaches −40 mV, the α1c subunit undergoes a conformational change, allowing calcium to be conducted from the extracellular environment into the cytosol [75]. The α2 and δ subunits are situated extracellularly and play a role in the anchoring and transport of the channel to the plasma membrane [75]. The β subunit is localized intracellularly bound to the I-II linker of the α1c subunit through the alpha interaction domain (AID) [75,76]. The β subunit facilitates channel trafficking to the plasma membrane and influences the inactivation properties of the channel [75,76].

#### 4.1.1. Current Therapeutics Targeting the L-Type Calcium Channel

Current therapies that target the L-type calcium channel for the treatment of heart disease are L-type calcium channel antagonists that block calcium conductance through the α1c subunit [34,77]. There are different classes of L-type calcium channel antagonists including phenylalkylamines (e.g., verapamil), dihydropyridines (e.g., nifedipine), and benzothiazepines (e.g., diltiazem). Phenylalkylamines and benzothiazepines both directly block calcium conduction by binding to residues inside the pore of the α1c subunit [78,79,80]. Dihydropyridines block calcium conductance allosterically by preventing channel opening, bringing S5_III_ and S6_IV_ closer to each other [78,81,82,83,84]. This interaction is proposed to lock the channel in the inactivated state by restricting conformational changes in the α1c subunit [78]. L-type calcium channel antagonists are used to treat cardiomyopathies as they block Ca^2+^-induced Ca^2+^ release and subsequently restrict Ca^2+^ uptake by the sarcomere reducing heart rate and contractility to improve diastolic filling. Due to the negative ionotropic effects of the L-type calcium channel antagonists [85], caution must be taken when prescribing these drugs, as they can decrease ejection fraction, resulting in poor clinical outcomes.

Another US Food and Drug Administration-approved drug that has been recently reported to influence the L-type calcium channel is semaglutide. Semaglutide is a glucagon-like peptide-1 (GLP-1) receptor agonist that is prescribed to patients for obesity and HFpEF. As a GLP-1 receptor agonist, semaglutide activates GLP-1 receptors augmenting insulin secretion, inhibits glucagon release, and suppresses gluconeogenesis [86,87]. However, semaglutide can also protect against the development of cardiac hypertrophy and heart failure by reducing L-type calcium channel current, Ca^2+^ transients, and consequently the contractility of the cardiac myocyte [88]. As semaglutide treatment does not alter Ca^2+^ handling genes [88], it is proposed that semaglutide’s regulation of the L-type calcium channel is through post-translational modifications.

The exact mechanism associated with semaglutide’s post-translational modification of the L-type calcium channel remains unclear. However, Sequeira V et al. propose that this could be through its action on the fatty acid palmitoyl. Palmitoyl has been reported to activate the L-type calcium channel and Ca^2+^ uptake in cardiac myocytes. Furthermore, high-fat diets that contribute to obesity result in an increase in the uptake and concentrations of fatty acids such as palmitoyl. These fatty acids accumulate in the cardiac myocyte, contributing to the development of HFpEF [88]. Therefore, the efficacy of semaglutide in HFpEF may be due to indirect effects on fatty acid uptake and subsequent L-type calcium channel post-translational modifications [88]. Further work is needed to clarify how semaglutide can alter post-translational modification of the L-type calcium channel to further evaluate the efficacy of semaglutide in HFpEF.

#### 4.1.2. Novel Therapeutics Targeting the L-Type Calcium Channel

**Figure 3:**
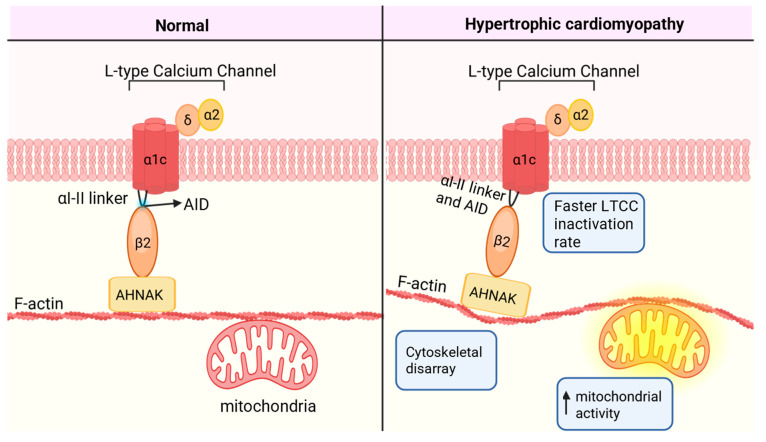
Alterations in the structural–functional coupling of the L-type calcium channel, the cytoskeleton, and mitochondria evident in hypertrophic cardiomyopathy. The β subunit of the L-type calcium channel regulates the inactivation properties of the channel and is coupled to the cytoskeleton through interactions with AHNAK. In hypertrophic cardiomyopathy, the presence of cytoskeletal disarray couples with an alteration in the inactivation rate of the L-type calcium channel, leading to an alteration in the communication between the L-type calcium channel and the mitochondria, which contributes to a hypermetabolic state and the development of hypertrophic cardiomyopathy. Abbreviations: AID = alpha interaction domain. AHNAK = neuroblast-associated differentiation protein.

Despite the clinical use of L-type calcium channel antagonists and semaglutide in the treatment of HFpEF and diastolic heart disease, there is an unmet need for novel therapeutics that target the mechanisms causing diastolic heart disease. Although the L-type calcium channel is canonically known to conduct calcium into the cardiac myocyte, many cardiomyopathies do not involve altered calcium conductance. Rather, it has been proposed that the interaction between the L-type calcium channel and the cytoskeleton is altered in disease [11,89,90,91,92]. In hypertrophic cardiomyopathy, the cardiac myocytes exhibit significantly faster L-type calcium channel inactivation rates compared to wildtype myocytes, cytoskeletal disarray, and a hypermetabolic state [90,93] (Figure 3). Conversely, in Duchenne Muscular Dystrophy, the lack of the intermediate filament dystrophin results in slower L-type calcium channel inactivation rates and a hypometabolic state [91,94]. These reports provide evidence that altered mitochondrial function in cardiomyopathies can be a result of a structural–functional breakdown between the L-type calcium channel and the mitochondria, mediated by the cytoskeleton (Figure 3). Therefore, the interactions between the L-type calcium channel, the cytoskeleton, and the mitochondria are potential novel therapeutic sites to treat diastolic heart disease.

The β subunit of the L-type calcium channel is responsible for the inactivation of the channel [76]. In fact, a fast inactivation rate is evident in hypertrophic cardiomyopathy [90,93] (Figure 3). Owing to the relationship between altered inactivation of the L-type calcium channel in hypertrophic cardiomyopathy, the interaction between the α1c and β subunits has been identified as a novel therapeutic target to treat diastolic dysfunction. The α1c and β subunits interact via the alpha interaction domain (AID). A peptide directed against the AID region (AID-TAT) can disrupt the interaction between AID and the β subunit, prolonging channel inactivation [95]. The acute application of the AID-TAT peptide to isolated ventricular cardiomyocytes isolated from hypertrophic cardiomyopathy mice, or administration of the AID-TAT peptide via intraperitoneal injection in *cTnI-G203S* hypertrophic cardiomyopathy mice, can alleviate the hypermetabolic state [95]. Furthermore, the cytoskeleton is in disarray in hypertrophic cardiomyopathy, which is proposed to contribute to the hypermetabolic activity of the mitochondria. Treatment of mice with the AID-TAT peptide for 5 weeks can correct the F-actin disarray in the *cTnI-G203S* myocytes, mimicking the arrangement seen in wildtype cells [95]. Hence, targeting the AID region of the β subunit alters the structural–functional link between the L-type calcium channel and mitochondria.

The AID-TAT peptide and variants of the AID-TAT peptide have been investigated in the prevention of hypertrophic cardiomyopathy in two preclinical mouse models of hypertrophic cardiomyopathy [89,95]. Both the AID-TAT peptide and the AID-TAT variants were able to prevent the development of hypertrophy in *cTnI-G203S* and *αMHC^403/+^* (mouse carrying in MYH7 hypertrophic cardiomyopathy mutation in MHY6) mice, as evidenced by improvements in contractility on echocardiography and decreased heart weight–tibial length ratios [89,95]. The AID variants were also able to improve left ventricular outflow tract (LVOT) pressures, prevent the development of fibrosis, and normalize mitochondrial function to wildtype levels [89,95]. This demonstrates that disrupting the structural–functional association between the L-type calcium channel and the mitochondria by targeting proteins involved in linking the L-type calcium channel to the cytoskeleton can prevent the development of hypertrophy and fibrosis in the context of hypertrophic cardiomyopathy.

The interaction between the L-type calcium channel β subunit and the actin cytoskeleton is established through the large scaffolding protein neuroblast-associated differentiation protein AHNAK, also known as desmoyokin [96]. AHNAK interacts with the β subunit via the C-terminal domain [97]. The structural coupling of the β subunit and F-actin through AHNAK plays a crucial role in regulating the transduction of signals between the L-type calcium channel, cytoskeleton, and mitochondria [11,96]. Disruption of the interactions between the β subunit and AHNAK with the peptide AHNAK-P4-TAT attenuated the hypermetabolic state induced by L-type calcium channel activation in myocytes plated on stiff hydrogels [11]. Hence, the structural–functional coupling of the L-type calcium channel to the cytoskeleton via AHNAK is proposed to influence both L-type calcium channel and ECM-mediated alterations in mitochondrial function. This reinforces the importance of the cytoskeletal link between the ECM, L-type calcium channel, and mitochondria and how dysregulation results in disease. Targeting the structural functional connection sites between the L-type calcium channel and mitochondria is a promising therapeutic strategy for diastolic heart disease.

#### 4.1.3. Translational Considerations

The translation of novel therapeutics that target the L-type calcium provides a promising therapeutic strategy that targets the communication between the L-type calcium channel, ECM, cytoskeleton, and the mitochondria. However, these studies have currently only been investigated in preclinical animal models and are limited to the prevention of hypertrophy in hypertrophic cardiomyopathy. Replication of the preclinical studies in large preclinical animal model cohorts and in human clinical trials is required to elucidate whether disrupting the interaction between AID and the β subunit of the L-type calcium channel is required to translate the AID-TAT-variant peptides into the clinic.

Another translational consideration is the mode of administration of the AID-TAT variants. Current studies utilize a cell-permeable-TAT sequence attached to the AID-variant peptides, which requires an injectable administration route. Patient adherence to therapeutics can be influenced by the administration route of the drug. Patients are more likely to adhere to a treatment regime which has an oral formulation compared with an injectable formulation. Therefore, future work should investigate how to deliver the AID variants either via an oral formulation or via inhalation using nanoparticles.

Studies of AID-variant peptides have also exclusively been carried out in the context of hypertrophic cardiomyopathy. Whether the AID-TAT peptide and its variants can be applied to other causes of diastolic heart disease has yet to be investigated. The AID-TAT peptide does display potential in treating other forms of diastolic heart disease, as the AID-TAT peptide can attenuate the hypermetabolic state of wildtype myocytes plated on stiff hydrogels [11]. Disruption of the interaction between the L-type calcium channel and the cytoskeleton was sufficient to disrupt ECM-induced increases in mitochondrial function, signifying the importance of the link between the L-type calcium channel and the cytoskeleton in mediating the maladaptive signaling between the ECM and the mitochondria. As AID-TAT can disrupt the maladaptive feedback that contributes to a hypermetabolic state, the AID-TAT peptide may be effective in treating diastolic dysfunction in pathologies involving ECM remodeling and stiffening.

### 4.2. Mechanosensitive Channels: PIEZO Channels, TRPM Channels

Piezo1 and Piezo2 ion channels are members of the recently discovered Piezo channel family, which play a crucial role in cellular mechanotransduction—the process by which mechanical stimuli are converted into cellular signals [98,99,100,101]. These mechanoreceptor membrane proteins are responsible for detecting extra- and intracellular mechanical cues and are involved in numerous physiological processes in eukaryotic organisms, including touch sensation, proprioception, vascular development, and regulation of blood pressure. Because of the significant role that Piezo1 and Piezo2 play in a variety of physiological processes and the impact their activation or deletion can have on mechanically driven cellular signaling in humans, discovery of these molecular mechanoreceptors was in 2021 recognized by the Nobel Assembly in Karolinska with the award of one half of the Nobel Prize for Physiology or Medicine to Ardem Patapoutian and his team at the Scripps Research Institute [102,103]. Beyond the ground-breaking Piezo channel work, this prize also recognizes the essential role that mechanical force, in all its forms, plays in living organisms and affirms the potential of both channels as targets for the development of new drugs for future treatments of a wide range of mechanochannelopathies.

**Figure 4:**
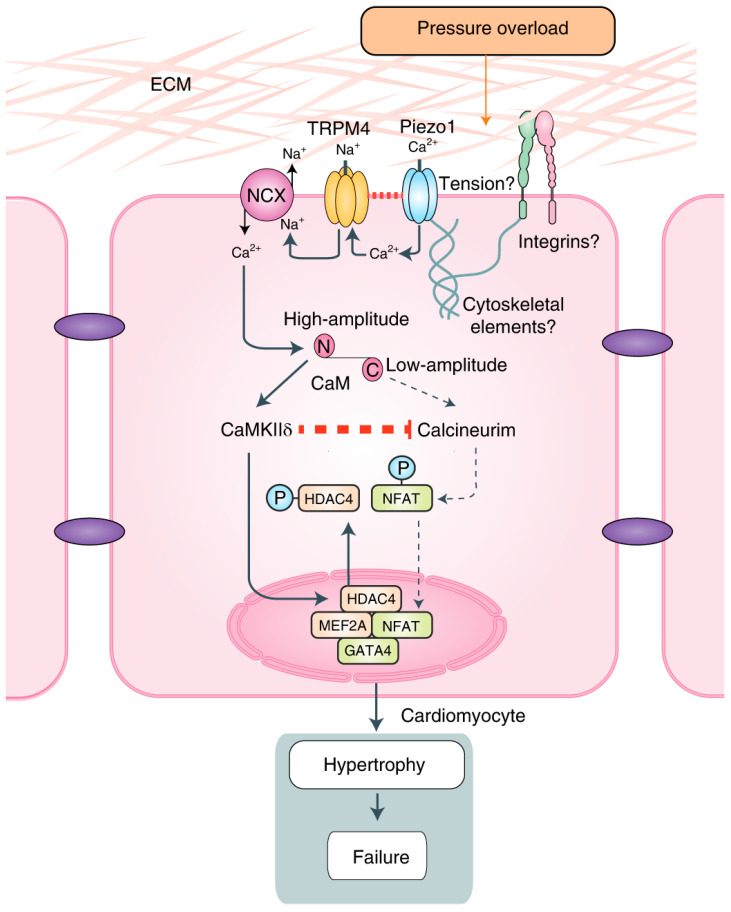
Schematic of the Piezo1-dependent signaling pathway that drives left ventricular hypertrophy secondary to pressure overload. Piezo1 acts as a mechanosensor in cardiomyocytes and initiates Ca^2+^ entry into cardiomyocytes upon pressure overload. The increase in local [Ca^2+^] results in activation of the TRPM4 channel, which is central to activation of a Ca^2+^-/CaM-dependent kinase II (CaMKII)-dependent hypertrophic signaling pathway after left ventricular pressure overload. Increase in TRPM4-dependent local [Na^+^] drives the Na^+^/Ca^2+^ exchanger (NCX) to extrude Na^+^, resulting in less extrusion of Ca^2+^ or Ca^2+^ entry via NCX, which results in a high-amplitude increase in local [Ca^2+^]. Acting as a bipolar switch, calmodulin responds to this high-amplitude Ca^2+^ stimulus through the lower-affinity Ca^2+^ binding sites at its N-lobe, which preferentially activates CaMKIIδ kinase and thus the CaMKII-HDAC4-MEF2 pathway to induce left ventricular hypertrophy. In contrast, the calcineurin–NFAT signaling pathway is activated preferentially by low-amplitude Ca^2+^ signaling via Gq-coupled receptors and calmodulin. In addition, calcineurin activation is strongly inhibited by activated CaMKIIδ. These two mechanisms explain the strong functional segregation of the two hypertrophic signaling pathways despite their common dependence on activation via Ca^2+^/calmodulin. ECM, extracellular matrix; CaM, calmodulin. The arrows indicate the directions of the signaling pathways. (Reproduced with permission from Yu et al. [108]).

As a discontinuous electromechanical pump, the human beating heart is adapted to large biomechanical stresses by performing on average some 100,000 beats per day, underlying its pumping capacity of about 7000 L of blood through the body [104]. There is substantial emerging evidence for the Piezo1/2 channel function in cardiovascular systems where these mechanosensitive membrane proteins contribute to endothelial shear stress sensing, regulation of vascular tone as well as vascular permeability, remodeling and development, blood pressure regulation, and the baroreceptor reflex. Furthermore, recent evidence indicates their relevance to cardiac fibroblasts and myocytes, suggesting the Piezo1 channel is a strong candidate contributing to cardiac mechanoelectric feedback through its Ca^2+^ transient regulation during cardiac cell stretching [105,106,107]. This notion found strong support in a recent study showing that Piezo1 functions as a cardiac mechanoreceptor at the origin of the intracellular Ca^2+^/Calmodulin-dependent kinase II (CaMKII)–histone deacetylase (HDAC) 4–myocyte enhancer factor 2 (MEF2) signaling cascade that initiates left ventricular hypertrophy along with fibrosis in response to cardiac pressure overload (Figure 4) [108]. Significantly, Piezo1 activation initiates hypertrophic signaling via close physical interaction with the TRPM4 ion channel, which plays a central role in amplifying the initial Ca^2+^ signal provided by Piezo1 as the primary mechanoreceptor [109,110].

Several other studies linked Piezo1 channel activity to different cardiomyopathies. The study by Jiang et al. indicates that Piezo1 mediates mechano-chemo-transduction in the heart by converting stretching cardiomyocytes during diastolic filling into Ca^2+^ and ROS signaling. Another study by Zhang et al. [111] suggests that hypertension-induced pressure overload in the heart leads to pathological cardiac hypertrophy. Although both studies provide further solid evidence for the involvement of Piezo1 channels in cardiac pathologies, the interpretation of the results that these studies provide raises more questions than they answer. The proposed molecular pathways to explain the described cardiac pathology results are only partially correct because the essential role of calmodulin in the corresponding signaling pathways was apparently overlooked. Calmodulin functions as a bipolar switch in the activation of calcineurin–Nuclear Factor of Activated T-cells (NFAT) and CaMKII-HDAC4-MEF2 pathways during diastolic filling, stretching the left ventricle and systolic ventricular contraction, respectively. Due to its molecular structure, calmodulin distinguishes between high-amplitude and low-amplitude Ca^2+^ signals, given the differential Ca^2+^ sensitivity of its C- and N-lobes [112,113,114] that enables differential activation of the calcineurin-dependent and CaMKII-dependent hypertrophic signaling pathways, respectively. The high-amplitude Ca^2+^ stimulus occurring during systolic cardiomyocyte contraction, which stretches T-tubules of cardiomyocytes where Piezo1 is localized, activates calmodulin through the lower-affinity Ca^2+^ binding sites at its N-lobe, followed by activation of the CaMKII-HDAC4-MEF2 pathway, which leads to cardiac hypertrophy (Figure 4). Importantly, once activated, CaMKII inhibits calcineurin activation [115]. Consequently, unlike voltage-gated Ca^2+^ channels localized in T-tubules, Piezo1 does not seem to contribute to the basal level of Ca^2+^ within cardiomyocytes but supplies increased Ca^2+^ transients during systolic contraction of the left ventricle.

Concerning the role of the Piezo1 channels in cardiac physiology, a recent report suggests a link between Piezo1 activity and aging [116]. The results of the study indicate that conditional deletion of Piezo1 from cardiomyocytes in adult mice resulted in premature mortality due to altered cellular Ca^2+^-handling kinetics causing bradycardia and cardiac fibrotic remodeling in the aging right atrium. Thus, in addition to its role in cardiac cardiomyopathies, Piezo1 emerges as a homeostatic molecular mechanosensor playing a role in the adaptation to an aging cardiac tissue. This provides a plausible explanation for the differential function of Piezo1-facilitated Ca^2+^ influx under physiological compared to pathological conditions.

These studies demonstrate a critical role of the Piezo1 channels in cardiac mechanotransduction and the downstream Ca^2+^-dependent signaling in heart physiology and pathology. The findings also suggest Piezo1 and associated signaling molecules, such as TRPM4, as potential targets for the development of novel therapies to treat mechano-pathologies involving Piezo1 channels.

#### 4.2.1. Therapeutic Strategies Targeting Piezo1 Channels

Piezo1 channels are found throughout non-sensory tissues. They are particularly highly expressed in the skin, lung, bladder, and gastrointestinal tract [117,118,119]. Irrespective of their different expression levels in various tissues, they are emerging as a promising therapeutic target for a variety of diseases, including cardiovascular diseases, cancer, and neurological disorders. Hence, the focus of one of the current key research areas in the mechanotransduction field is to search for compounds that either specifically activate or inhibit Piezo1 channel function, with progress being made in understanding small-molecule modulators of Piezo1. For example, activators like Yoda1 and Jedi1/2 are capable of opening Piezo1 ion channels in the absence of mechanical stimulation. Jedi1/2 functions through a unique peripheral blade mechanism of the channel to gate the ion-conducting pore, while Yoda1 lowers the activation threshold of Piezo1 by inducing conformational changes. Additionally, non-specific inhibitors such as gadolinium and ruthenium red can block Piezo1 channels, and the spider toxin GsMTx4 inhibits these channels by altering local membrane tension rather than binding directly [120,121]. Since none of these compounds are currently suitable for clinical applications in the treatment of Piezo1 channelopathies, the search for alternative novel strategies is continuing.

Recent studies demonstrated that mammalian transient receptor ion channels (TRPs) are not activated by membrane stretch [122] but rather act in concert with primary mechanotransducers, such as Piezo1 channels, phospholipase A2 (PLA2), or G-protein-coupled receptors (GPCRs) to amplify, integrate, or modify the mechanical signal [110]. These findings seem to offer new possibilities to target Piezo1 mechanochannelopathies. The above-discussed Piezo1-TRPM4 channel interaction in the process of left ventricular hypertrophy may serve as a case in point. A recent report in *Science* showed that inhibiting physical coupling between the N-methyl-D-aspartate receptor (NMDAR) channel and TRPM4 strongly reduced NMDAR-triggered Ca^2+^ toxicity in mouse brains [123]. Thus, the finding of a similar interaction between Piezo1 and TRPM4 (Figure 4) opens an attractive possibility of developing novel compounds targeting specifically the Piezo1/TRPM4 interface for treatment of cardiac hypertrophy and possibly other cardiac mechanochannelopathies.

#### 4.2.2. Clinical Evidence

There is currently no direct clinical evidence of treatments for cardiac diseases, including HFpEF, that are specifically caused by the aberrant activity of Piezo1 channels. However, given that Piezo1 channels play a significant role in the healthy as well as diseased heart, developing new compounds to target them presents a promising therapeutic avenue.

#### 4.2.3. Translational Considerations

Developing drugs that target Piezo1 channels in specific organs or tissues is not a simple task given the involvement of the channel in essential mechanotransduction processes in organ systems throughout the body. Targeting Piezo1 channels alone might lead to severe side effects due to their broad expression. To illustrate this, Li et al. demonstrated that global or endothelial-specific deletion of Piezo1 in mice was embryonically lethal, profoundly affecting the development of vasculature [106]. Furthermore, targeting Piezo1 ion channels with pharmacological agents poses significant risks of serious side effects due to their critical roles in various physiological processes across numerous tissues and organs, including cardiovascular, musculoskeletal, and nervous systems. Since Piezo1 functions as a mechanosensor essential for processes such as blood pressure regulation, bone development, and pain perception, any disruption in its activity could lead to widespread and unpredictable consequences, such as hypertension, impaired bone formation, or altered pain signaling [124]. Also, the dual role of Piezo1 in cancer where it is acting both as a promoter and suppressor of tumors adds to the complexity, rendering the outcomes of targeting this channel highly uncertain [125]. Moreover, the intricate interactions that the Piezo1 channel shares with other cellular components further complicate drug interventions, highlighting the need for meticulous research and evaluation to mitigate potential adverse effects before pursuing therapeutic strategies aimed at this channel [126]. Consequently, while Piezo1 is a promising target for certain diseases, its widespread expression and involvement in many physiological processes mean that any drug intervention must be carefully designed and evaluated to minimize the risk of serious side effects.

## 5. Drug Target: Sarcomere

The sarcomere is a multiprotein complex responsible for the generation of active and passive force in the cardiac myocyte [127]. Myosin and actin filament sliding generates an active force, shortening the sarcomere, which results in the pumping of blood from the heart into the vascular system [127,128]. Passive force is regulated by titin, which facilitates passive ventricular filling [128]. Alterations in the structure and function of sarcomere proteins and sarcomere regulatory proteins contribute to the establishment of diastolic dysfunction. Often, these alterations increase contractility, alter myofilament Ca^2+^ sensitivity, and alter passive tension. Genetic mutations in the sarcomere or regulatory proteins contribute to hypertrophic cardiomyopathy by inducing a hypercontractile state and alter myofilament Ca^2+^ sensitivity [129,130,131]. Alterations in sarcomere ultrastructure and passive tension have been reported in diabetic cardiomyopathy and HFpEF [131,132]. Considering diastolic dysfunction involves alterations in the structure and function of the sarcomere, the sarcomere and its regulatory components are potential therapeutic targets to treat diastolic dysfunction. In this section, we will describe how the different components of the sarcomere contribute to diastolic heart disease and discuss the efficacy of current and novel therapeutic targets.

### 5.1. Myosin (Thick Filament)

Myosin is a major component of the sarcomere involved in force generation and contractility. Hence, it is not surprising that mutations affecting myosin are well characterized as causes of cardiomyopathies. It is estimated that ~83% of hypertrophic cardiomyopathy patients exhibit mutations in the myosin-encoding genes MYBPC3 and MYH7 [133]. In the context of hypertrophic cardiomyopathy, myosin mutations contribute to excessive sarcomere force generation and hypercontractility [129]. Hypercontractility and altered sarcomere ultrastructure are also evident in HFpEF and diabetic cardiomyopathy [128,132]. The hyperdynamic properties of the mutant sarcomeres are proposed to contribute to the downstream development of hypertrophy and to a maladaptive feedback loop between the cardiac cytoskeleton and ECM. Hence, there has been an emphasis on treating hypercontractility by developing novel therapeutics that target myosin.

#### 5.1.1. Therapeutic Strategies Targeting Myosin

Mavacamten is a US Food and Drug Administration-approved small-molecule inhibitor of myosin ATPase currently prescribed for hypertrophic cardiomyopathy [34,77]. The binding of mavacamten to the myosin S1 region inhibits the cardiac myosin catalytic domain, reducing the rate of inorganic phosphate release from the myosin head without impacting adenine diphosphate (ADP) release [134]. As inorganic phosphate release is the rate-limiting step in sarcomere contraction, reducing inorganic phosphate release limits the ability of the myosin head to bind actin, reducing sarcomere-generated force and contractility [134]. Wildtype and *αMHC^403/+^* mice exhibit a reduction in fractional shortening with mavacamten treatment [134]. A reduction in LVOT gradient was also evident in cats with obstructive hypertrophic cardiomyopathy following treatment with mavacamten [135]. Furthermore, mavacamten was able to disrupt the development of fibrosis in *αMHC^403/+^* mice when treated with mavacamten prior to the development of hypertrophic cardiomyopathy [134]. These reports emphasize the role of the hypercontractile sarcomere in the development of hypertrophy, fibrosis, and hypertrophic cardiomyopathy progression. By normalizing the hyperdynamic state, mavacamten can ameliorate the pathology and disrupt the maladaptive feedback loop.

Another small-molecular inhibitor currently progressing through clinical trials is aficamten. Like mavacamten, aficamten binds to myosin S1 and is a direct inhibitor of the cardiac myosin catalytic domain [136]. However, the binding site of aficamten differs from mavacamten, as aficamten shares a binding site with the non-specific myosin inhibitor blebbistatin [136]. Aficamten can inhibit cardiac myosin by binding close to the inorganic phosphate release door, which reduces the rate of inorganic phosphate release and consequently weakens the binding affinity between myosin heads and the actin filaments [136]. In cardiac myofibrils, aficamten application reduced fractional shortening in a dose-dependent manner [136]. A reduction in fractional shortening was also evident in healthy rat, mouse, and dog animal models as well as in *αMHC^403/+^* mice [136].

#### 5.1.2. Clinical Evidence

In the clinic, mavacamten is prescribed to patients with severe obstructive hypertrophic cardiomyopathy. Mavacamten treatment improves the LVOT gradient in patients, alleviating symptoms such as dyspnea and improving exercise tolerance [137,138]. Improvements in the LVOT gradient have also reduced the number of patients requiring invasive septal myectomy surgery [139]. However, obstructive hypertrophic cardiomyopathy encompasses two-thirds of hypertrophic cardiomyopathy patients [140]. Mavacamten has yet to be administered for the treatment of non-obstructive hypertrophic cardiomyopathy despite the risk of these patients progressing to an obstructive phenotype. There is also a lack of evidence for a clinical reduction in cardiac hypertrophy. Similar clinical findings have been reported with afiacamten [141]. Therefore, further work needs to be carried out to assess whether mavacamten can be used in less severe hypertrophic cardiomyopathy and to identify other therapeutic targets that can reverse the development of cardiac hypertrophy.

The efficacy of mavacamten outside the context of hypertrophic cardiomyopathy has only recently been explored in the EMBARK-HFpEF clinical trial. A 26-week treatment with 2.5–5 mg of mavacamten significantly reduced circulating NTproBNP, hsTnT, and hsTnI and significantly improved diastolic function as reported by E/e’ and left atrial volume [142]. Mavacamten treatment caused a reduction in ejection fraction from 66.82% to 63.95% in HFpEF patients, but this is not clinically significant as the reduction does not indicate the development of systolic dysfunction [142]. Overall, the preliminary findings reported in the EMBARK-HFpEF trial provide promising evidence towards the use of mavacamten, and potentially other cardiac myosin inhibitors, for HFpEF.

#### 5.1.3. Translational Considerations

The translation of mavacamten and afiacamten into the broader context of diastolic heart disease has some important considerations that must be addressed. Firstly, the only clinical evidence for the efficacy of mavacamten in HFpEF is derived from an open-label study with a small cohort. To translate the beneficial findings of the EMBARK-HFpEF trial into the clinic, further studies in larger randomized double-blinded cohorts must be carried out. Similar clinical trials should also be considered for afiacamten, considering the success of afiacamten in clinical trials for hypertrophic cardiomyopathy.

Another consideration that should be investigated is the mechanistic benefit of myosin inhibitors in the context of HFpEF. Small myosin inhibitors have been successful in the context of hypertrophic cardiomyopathy, as myosin mutations contribute to the development of cardiac hypertrophy. Myosin mutations make up a considerable proportion of the hypertrophic cardiomyopathy patient cohort; however, other mutations such as troponin I and troponin T mutations can also result in hypertrophic cardiomyopathy [133]. Studies have reported a reduced efficacy of mavacamten in cardiac myocytes derived from inducible pluripotent stem cells containing troponin T mutations [143]. As HFpEF is a result of a diverse range of contributing factors involving structural and metabolic alterations, the efficacy of mavacamten and other small myosin inhibitors may be reduced. However, as mavacamten can relax the myocardium, combining mavacamten with therapeutic strategies that target the metabolic abnormalities in HFpEF may enhance the efficacy of mavacamten in patients with metabolically induced diastolic dysfunction.

### 5.2. Thin Filament (Actin, Tropomyosin, and the Troponin Complex)

The thin filament is a complex of proteins including F-actin, the troponin complex (troponin C, troponin I, and troponin T), and tropomyosin. Contraction of the sarcomere is controlled by the thin filament’s troponin complex and tropomyosin [144]. At resting Ca^2+^ levels, the troponin complex anchors tropomyosin to actin, preventing the interaction between actin and myosin [144]. An increase in intracellular Ca^2+^ upon release of Ca^2+^ from the sarcoplasmic reticulum results in Ca^2+^ binding to troponin C and the subsequent release of the troponin complex and tropomyosin from actin [144]. The release of tropomyosin exposes the myosin head binding sites on the actin filament, allowing myosin head binding and contraction [132].

Mutations in the troponin complex have been identified as causes of hypertrophic cardiomyopathy, and increased myofilament sensitivity to Ca^2+^ is evident in diastolic heart disease and metabolic syndrome [90,133,144,145,146]. However, although alterations in the thin filament and troponin complex have been associated with pathology, novel therapeutics that target the thin filament remain limited. Preclinical investigations predominantly investigate drugs that alter Ca^2+^ sensitivity by targeting troponin C [147,148,149] or target the phosphorylation of troponin I [150,151]. However, despite the preclinical investigations, there are currently no drugs that target the thin filament that are US Food and Drug Administration-approved to treat diastolic heart disease.

#### 5.2.1. Therapeutic Strategies Targeting the Thin Filament

Levosimendan is a positive inotropic drug that is clinically approved for use in some European and South American countries that binds troponin C to sensitize myofilaments to calcium, acts on K_ATP_ channels to dilate arteries, and inhibits phosphodiesterase-3 [152]. Due to the positive inotropic effects of levosimendan, it has predominantly been investigated in the context of systolic dysfunction, such as acute decompensated heart failure and dilated cardiomyopathy. As systolic function is preserved in HFpEF, levosimendan’s efficacy would theoretically be limited. However, preclinical HFpEF animal studies suggest otherwise.

Reports from two preclinical murine models of HFpEF suggest that levosimendan may be effective for the treatment of HFpEF. Levosimendan treatment improved diastolic function in Zucker fatty and spontaneously hypertensive (ZSF-1) obese rats and the two-hit (high-fat diet and L-NAME administration) HFpEF C57BL/6N mice [147,148,149]. Furthermore, levosimendan treatment attenuated left ventricular hypertrophy and myocardial fibrosis whilst preserving left ventricular ejection fraction [147,148,149]. Treatment of ZSF-1 obese rats with levosimendan resulted in a downward shift in the sarcomere length–passive tension relationship [147], suggesting the treatment was able to enhance diastolic compliance. It is proposed that levosimendan’s efficacy in HFpEF could be attributed to a reduction in afterload and attenuation of markers of endothelial dysfunction, mitochondria dysfunction, and ferroptosis. Interestingly, systolic function was not further enhanced with levosimendan treatment, and this finding was also evident in patients with pulmonary hypertension [147,149,153]. These findings suggest that levosimedan may be effective in the treatment of diastolic dysfunction.

Phosphorylation of troponin I has been implicated in the regulation of myofilament Ca^2+^ sensitivity [144]. Troponin I can be phosphorylated by protein kinase A (PKA) at serine 23/24 (S23/24) or by the Src tyrosine kinase at tyrosine 26 (Y26), reducing myofilament Ca^2+^ sensitivity and enhancing relaxation [150,151]. Reduced troponin I phosphorylation is evident in HFpEF, suggesting that troponin I phosphorylation could be a possible therapeutic target for diastolic dysfunction. Preclinical mouse models have demonstrated that phosphorylation of either S23/24 or Y26 can protect against diastolic dysfunction without impairing systolic function [150,151]. Considering the important contribution of myofilament sensitivity in diastolic function, targeting S23/24 and Y26 troponin I phosphorylation may be a potential therapeutic target that can relax the heart and disrupt the maladaptive feedback between the cytoskeleton and the ECM.

#### 5.2.2. Clinical Evidence

The clinical evidence for targeting the thin filament involves the troponin complex. Levosimendan acts on troponin C, altering myofilament sensitivity, and was investigated in a cohort of patients with pulmonary hypertensive HFpEF. A 6-week treatment with levosimendan did not significantly alter cardiac function (LV global longitudinal strain, RV free wall strain, tricuspid annular plane systolic excursion, or left atrial conduit strain) [153]. Despite being a positive inotropic drug, levosimendan did not significantly alter ejection fraction after the 6-week treatment period [153]. The lack of cardiac parameter improvement suggests that levosimendan is not effective at treating the cardiac complications associated with pulmonary hypertensive HFpEF and, therefore, may not be suitable for other HFpEF subtypes.

A clinical trial investigating the therapeutic benefit of PKA phosphorylation of troponin I failed due to the off-target phosphorylation of other cardiac proteins, resulting in arrythmias and increased mortality [154]. The effects of Src tyrosine enhancement on phosphorylate troponin I have yet to be identified in human cohorts. However, some preclinical models indicate that c-Src tyrosine kinase activation can result in arrythmias by reducing connexin-43 expression [155]. As the evidence for the pharmacological targeting of troponin remains limited and maladaptive, the development of other troponin-targeted therapeutic strategies that exclusively target troponin should be pursued.

#### 5.2.3. Translational Considerations

Clinical trials involving levosimendan and PKA in the treatment of heart failure have highlighted translational considerations that should be addressed before progressing to the clinic. Firstly, the levosimendan clinical trial did not measure cardiac parameters as a primary outcome. Consequently, although echocardiographic measurements were acquired, there is a lack of HFpEF relevant measurements such as E/e’ which are clinically used to determine diastolic function. The study also consisted of a small sample size involving a subset of HFpEF patients that also exhibit pulmonary hypertension. To consider the clinical use of levosimendan for HFpEF, clinical trials should involve a larger cohort of HFpEF patients to identify the efficacy of levosimendan in general and in different subtypes of HFpEF. As levosimendan is also a positive inotropic drug, investigations into how levosimendan does not increase ejection fraction in the preclinical animal models and in the pulmonary hypertensive HFpEF patient cohort should be pursued.

The phosphorylation of troponin I is a promising therapeutic strategy for HFpEF. To translate this mechanism into a therapeutic target, studies should investigate how to overcome the deleterious effect of broad PKA activation. To achieve this, the identification of pharmacological agents that enhance troponin I phosphorylation without affecting the phosphorylation of other proteins in the heart must be considered.

### 5.3. Titin

Cardiac myocyte passive stiffness is primarily determined by titin particularly during diastole [156,157,158,159,160]. Titin also provides a stretch-resisting force that functions to restore the sarcomere to its resting length. The I-band is responsible for titin’s elasticity, supporting early diastolic recoil and late diastolic resistance to stretch. The elastic properties of the I-band involve the PEVK segment (a structurally disordered region rich in proline, glutamate, valine, and lysine), tandem Ig segments, and a cardiac-specific N2B unique (N2B-U) sequence flanked by Ig domains [161]. Isoform switching and post-translational modifications of the I-band contribute to alterations in cardiac myocyte stiffness in health and disease, making it a potential therapeutic target to treat diastolic dysfunction.

#### 5.3.1. Titin Isoform Switching as a Therapeutic Target

The two main isoforms of cardiac titin are N2B and N2BA [162]. The isoforms are co-expressed at an N2BA:N2B ratio of 30:70–40:60, which does not significantly change during aging [163]. Variations in the ratio of N2BA:N2B can regulate the passive mechanical behavior of the cardiac myocyte, altering the diastolic properties of the heart. An increase in the stiffer isoform, N2BA, is evident in HFpEF, HFrEF, and aortic stenosis [157,158,163,164,165,166,167]. Furthermore, the concentration of the circulating thyroid hormone T3, which influences cardiac contractility, can shift the ratio of N2B:N2BA [168]. As titin isoforms switch in disease, targeting titin isoform expression may be effective in the management of disease.

Investigations into titin isoform switching to treat diastolic dysfunction predominantly involve the RNA-binding motif protein 20 (RBM20), which regulates titin splicing [169]. A reduction in RBM20 protein results in the expression of long titin isoforms that are more compliant than shorter isoforms [169,170,171]. Reducing RBM20 expression with an antisense oligonucleotide that targets the 3′ untranslated region of the RBM20 mRNA has been investigated in mice with elevated diastolic stiffness, to identify if isoform switching can improve diastolic function. Indeed, the antisense oligonucleotide against RBM20 was able to improve diastolic function by upregulating the expression of longer and more compliant titin isoforms [172]. However, it has been reported that RBM20 also affects the splicing of *CAMK2D*, *LDB3*, and *CACNA1C*, and thus, the RBM20 antisense oligonucleotide should be used with caution as it could alter calcium handling [169].

#### 5.3.2. Post-Translational Modifications of Titin as a Therapeutic Target

Unlike isoform switching, which can take days to be effective, post-translational modifications are a fast way to alter titin’s passive stiffness. Post-translational modifications generally occur in the I-band, which controls titin’s elastic properties. Phosphorylation, S-glutathionylation, disulfide bond formations, and acetylation/deacetylation are post-translational modifications currently identified to alter titin stiffness. Of the post-translational modifications, phosphorylation is the most extensively researched.

The two phosphorylation hotspots of titin are the N2B-U element and the PEVK region [173,174,175]. N2B-U can be phosphorylated by guanosine 3′,-5′-cyclic monophosphate (cGMP)-dependent protein kinase G (PKG), CaMKIIδ, PKA, and the extracellular signal-regulated kinase 2 (ERK-2) [158,173]. Phosphorylation by any of these kinases at the N2B-U region reduces passive stiffness and enhances diastolic filling. PEVK phosphorylation occurs due to protein kinase C α (PKCα), CaMKIIδ, and protein kinase D. Unlike N2B-U phosphorylation, PEVK phosphorylation increases titin passive stiffness [158,174,175]. The opposing effects of I-band-region phosphorylation remain elusive; however, they are proposed to be due to structural alterations of titin. In diastolic dysfunction, titin-based stiffening is attributed to impaired cGMP-PKG signaling and increased PKCα activity, resulting in dysregulated titin phosphorylation early into disease progression [156,158].

As titin phosphorylation is well characterized in both health and disease, several therapeutics targeting titin phosphorylation have been investigated to treat diastolic dysfunction. Strategies to increase cGMP concentration by increasing PKG activity and reducing PKCα and CaMKII*δ* activity with either phosphodiesterase 5 inhibitors, phosphodiesterase 9 inhibitors [176], or soluble guanyl cyclase activators have been investigated in both preclinical animal models and in clinical trials [177]. Sildenafil, a phosphodiesterase 5 inhibitor, and soluble guanyl cyclase activators were able to reduce cardiac myocyte stiffness and increase left ventricular distensibility in preclinical animal models of diastolic dysfunction by increasing titin phosphorylation [177,178]. However, these drugs were unable to improve diastolic dysfunction in HFpEF patients [179,180,181]. Phosphodiesterase 9a inhibitors are currently undergoing clinical trials. In preclinical mouse models, phosphodiesterase 9a inhibitors were able to increase titin phosphorylation and reduce cardiac myocyte stiffness [176]. However, the authors noted that the doses required to achieve diastolic improvements impaired systolic function, making it an unattractive therapeutic agent for diastolic heart disease [176].

Diabetic cardiomyopathy is also associated with alterations in titin phosphorylation, and common diabetic drugs, such as metformin, have been identified to alter titin phosphorylation, enhancing diastolic compliance. Metformin is a first-line drug for the management of hyperglycemia in type II diabetes mellitus [182]. In clinical trials, metformin treatment reduces mortality by reducing cardiovascular risk [183,184,185]. Improvements in diastolic function have also been reported in the Metformin in Diastolic Dysfunction of Metabolic Syndrome clinical trial [186]. Metformin treatment of HFpEF mice (induced with transaortic constriction and deoxycorticosterone acetate pellet implantation) increased the phosphorylation of serine 4010 in the N2B-U region of titin [182]. Phosphorylation of this region reduced titin-based passive tension and was coupled with improvements in diastolic function, indicated by a reduction in the E/A ratio and E/e’ [182]. However, although diastolic function was improved with metformin treatment, left ventricular hypertrophy and ECM stiffness were unaffected [182]. This is consistent with the report that the positive effect of metformin on diastolic function was independent of cardiac hypertrophy [186].

Other post-translational modifications include S-glutathionylation, disulfide bond formation, and acetylation and deacetylation. Unlike phosphorylation, these modifications have been discovered more recently and have predominantly been investigated in preclinical animal models. As targeting titin phosphorylation has not been effective in the clinic, identifying novel therapeutics that target other post-translational modifications may be beneficial to treat diastolic dysfunction. Increased disulfide crosslinking in the N2B-U region increases titin passive stiffness [187]. Conversely, S-glutathionylation and deacetylation of the PEVK region with HDAC-6 have been reported to reduce titin passive stiffness [188,189].

#### 5.3.3. Translational Considerations

Preclinical animal models have demonstrated the positive effects of targeting titin compliance through either RBM20 or post-translational modifications. However, when translated to human clinical trials, these therapies fail to improve diastolic function (sildenafil and soluble guanyl cyclase) or improve diastolic function in the absence of reduced cardiac hypertrophy and fibrosis (metformin).

The complex nature of diastolic dysfunction may contribute to the lack of translatability of post-translational modification-targeting therapeutics. Under normal physiological conditions, titin is the main protein determining passive myocardial stiffness [157,165,190]. However, in disease, the paradigm shifts, and the passive stiffness of the heart is affected more by ECM stiffness than titin [191]. Hence, targeting titin alone may only be beneficial early in disease progression and may not be sufficient to improve well-established diastolic dysfunction. The diminished role of titin in controlling passive stiffness in well-established disease may be indicative of titin’s passive role in the maladaptive feedback loop, which contributes to diastolic dysfunction in advanced stages of HFpEF. Further work should investigate the use of a combination of therapies that target both titin phosphorylation and ECM stiffness to improve diastolic compliance.

## 6. Drug Target: Mitochondria

Mitochondria are organelles that produce adenine triphosphate (ATP) by glycolysis and oxidative phosphorylation. In cardiac myocytes, oxidative phosphorylation is the predominant form of ATP production [192]. ATP is essential for cardiac myocyte function, driving muscle contraction through the myosin head ATPase, enabling relaxation by acting on the sarco/endoplasmic reticulum calcium ATPase, and fueling other processes that maintain cellular homeostasis [127]. Owing to the importance of ATP hydrolysis in contraction, cardiac myocytes are densely packed with mitochondria, which are aligned along the sarcomere, allowing close localization of ATP production to the contractile apparatus.

Metabolic reprogramming is a hallmark feature of diastolic dysfunction, cardiomyopathies, and HFpEF. Increased ROS formation [192,193], mitochondrial protein hyperacetylation [6,192], abnormal mitochondrial calcium handling [194], and altered substrate handling (e.g., reduced phosphocreatine/ATP and NAD/NADH ratios) [195] are all components of the metabolic reprogramming that occur in diastolic dysfunction. Metabolic reprogramming and subsequent mitochondrial dysfunction are proposed to contribute to the development of fibrosis and altered contractile function [192], indicating that mitochondria may be central to the maladaptive feedback in diastolic heart disease.

### 6.1. Oxidative Stress and Reactive Oxygen Species

Oxidative stress is correlated with left ventricular dysfunction and hypertrophy in disease [196,197]. An excess of ROS induces oxidative stress by impacting subcellular organelles, altering enzymatic activity, inducing intracellular calcium overload, and regulating gene expression [192]. The imbalance between ROS production and antioxidants participates in the development and persistence of HFpEF, diabetic cardiomyopathy, hypertrophic cardiomyopathy, and diastolic dysfunction [198]. ROS can induce hypertrophic remodeling by activating hypertrophic signaling cascades and promoting post-translational modifications of cardiac proteins [197]. Proteins affected by excessive ROS include PKG [199], CaMKII [200], PKA [201], HDACs [202], MMPs [203], protein kinase B [204], ERK1/2 [197], PKC [205], and NK-kappa [206]. Therefore, ROS affects several pathways including protein acetylation, phosphorylation, calcium signaling, ECM remodeling, and hypertrophy. Of particular importance in diastolic dysfunction, many of the affected kinases are involved in titin phosphorylation, and thus, ROS can influence the passive stiffness of the cardiac myocyte. However, despite the well-established interplay between oxidative stress and diastolic dysfunction, targeting ROS production to improve diastolic function remains difficult.

#### 6.1.1. Therapeutic Strategies Targeting Oxidative Stress

As oxidative stress is associated with an imbalance between ROS and the antioxidant capacity of the cell, antioxidants are a potential therapy to reduce ROS levels in the heart. Mitoquinone is a mitochondrial antioxidant that has been investigated as a form of therapy to treat left ventricular dysfunction [207,208]. In a model of ascending aortic constriction, which rapidly develops cardiac fibrosis and left ventricular dysfunction, mitoquinone treatment attenuated hypertrophy, prevented left ventricular chamber remodeling, and reduced fibrosis in mice with aortic constriction [207]. The authors propose that mitoquinone’s antioxidant effects disrupted the positive maladaptive feedback loop involving TGF-β, NOX4, and redox signaling, which perpetuates cardiac fibrosis, hypertrophy, and ROS production [207]. Conversely, in rat models of pressure overload-induced heart failure (via aortic constriction) [208] or volume overload-induced heart failure (via aortocaval fistula) [209], mitoquinone was unable to improve diastolic dysfunction despite the preservation of mitochondrial function and partial restoration of cytoskeletal architecture. It is important to note that the three models are not ideal models to investigate diastolic heart disease, as systolic dysfunction and/or dilation was also present. Considering treatments for HFrEF have limited efficacy in HFpEF, studies using mitoquinone in the context of HFpEF and diastolic dysfunction need to be pursued to confirm whether targeting ROS formation with the application of mitoquinone can improve diastolic function in the absence of systolic dysfunction.

Elamipretide is a novel tetrapeptide that is associated with cardiolipin. Cardiolipin is a lipid embedded in the inner mitochondrial membrane that controls mitochondrial stability and the organization of respiratory complexes on the inner mitochondrial membrane [210,211,212]. As pathological alterations in respiratory complex activity contribute to excessive ROS production, stabilization of cardiolipin with elamipretide has been proposed to be beneficial in the treatment of oxidative stress. Indeed, the treatment of intracoronary microembolization-induced chronic heart failure dogs with elamipretide normalized complex-I and -IV activity and reduced ROS levels in the left ventricle [213]. The reduction in ROS was coupled with improvements in ejection fraction, left ventricular end diastolic pressure, cardiac hypertrophy, myocardial fibrosis, and myocardial ATP synthesis [213].

Dipeptidyl peptidase-4 inhibitors are prescribed to type 2 diabetic patients for hyperglycemic control but have also demonstrated cardioprotective effects in cardiac reperfusion injury [214,215], as well as improved atrial remodeling and diastolic dysfunction in diabetic rabbits [216,217]. In a diabetic mouse model that exhibits diastolic dysfunction, the dipeptidyl peptidase-4 inhibitor alogliptin was able to rescue the reduction in mitochondrial membrane potential and attenuate the increased ROS production evident in the diabetic mice [216]. Diastolic function was also preserved, and cardiac remodeling (hypertrophy and fibrosis) was prevented in the alogliptin-treated diabetic mice [216]. Similarly to mitoquinone treatment, alogliptin was able to prevent the increase in TGF-β expression in cardiac tissue [216]. Therefore, alogliptin’s antioxidant effects may disrupt the maladaptive feedback loop and pathological remodeling of the heart.

#### 6.1.2. Clinical Evidence

The well-established interplay between oxidative stress and cardiovascular disease has resulted in various clinical trials assessing the safety and efficacy of antioxidants in cardiac diseases. Although MitoQ has been evaluated in clinical trials, none of these trials has investigated the efficacy of MitoQ in the context of diastolic heart disease and heart failure. Elamipretide and alogliptin were promising in preclinical studies but lacked replication in cardiac functional improvements in the clinic [218,219,220]. The US Food and Drug Administration also advises against prescription of alogliptin to patients experiencing heart failure [218]. A study of the Memphis Veteran patient population reported that alogliptin exacerbated heart failure in 26% of patients with preexisting heart failure [218]. Due to the lack of strong clinical evidence supporting the use of antioxidants in heart failure, further work should be pursued to investigate why the translation of antioxidants from preclinical animal models to the clinic is hindered.

#### 6.1.3. Translational Considerations

Despite extensive research into the importance of oxidative stress in driving diastolic heart disease, therapeutic strategies targeting oxidative stress are not commonly prescribed in the clinic. This in part is due to the lack of translation between preclinical animal studies and human clinical trials. An understanding of why antioxidant-targeted therapies fail to transition into the clinic is of importance to develop future therapeutics targeting oxidative stress and ROS in diastolic heart disease.

One prominent barrier in the translation of antioxidant therapeutics into the clinic is the choice of preclinical animal models. For example, preclinical studies investigating the efficacy of MitoQ in heart failure use different models of heart failure. These models include two models that induced pressure overload heart failure (via ascending aortic constriction or aortic constriction) or volume overload-induced heart failure (aortocaval fistula). Although the phenotype of heart failure is established, the mechanisms that result in the establishment of heart failure are different and may contribute to the disparities in the results between the studies. Furthermore, these studies induced both diastolic and systolic failure, resembling HFrEF. Several studies establish the efficacy of the antioxidant in preclinical models resembling HFrEF. As HFpEF is distinct from HFrEF, it is important to evaluate the efficacy of antioxidants in robust preclinical animal studies of HFpEF before transitioning the therapies into clinical trials.

To overcome the diverse etiology of HFpEF, it may be beneficial to use antioxidants in a cocktail therapy. As the pathophysiology of HFpEF and diastolic heart disease involves a combination of oxidative stress, pathological remodeling of the ECM, altered mechanosensitive signaling through the costamere, and cytoskeletal stiffening combining antioxidants with treatments that target the ECM, the costamere or the cytoskeleton may improve the efficacy of antioxidants in the treatment of HFpEF.

### 6.2. Mitochondrial Protein Acetylation

**Figure 5:**
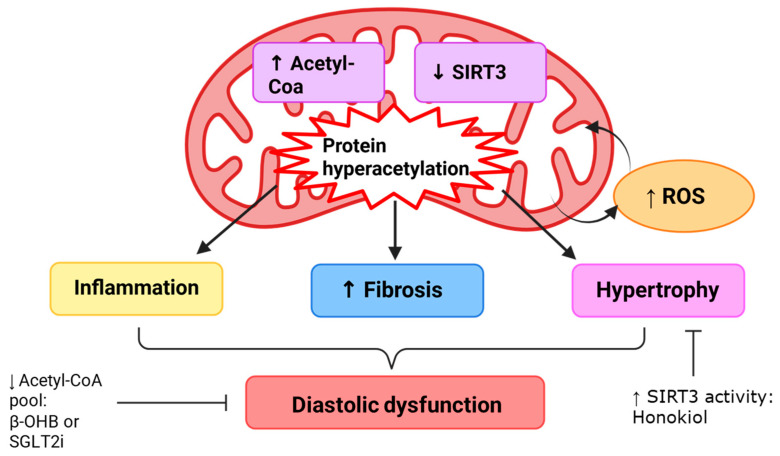
Protein hyperacetylation causes alterations in the cardiac myocyte which contribute to the development of diastolic dysfunction. An increase in the acetyl-CoA pool and/or a reduction in SIRT3 levels/activity results in protein hyperacetylation. Protein hyperacetylation contributes to the development and persistence of diastolic dysfunction by promoting inflammation, increasing fibrosis and hypertrophy, and increasing the level of ROS production as indicated by the arrows. Understanding the mechanisms associated with protein hyperacetylation in diastolic dysfunction provides potential therapeutic targets to treat diastolic dysfunction. For example, increasing SIRT3 activity with honokiol can prevent the development of hypertrophy, while reducing the acetyl-CoA pool, by either increasing ketone bodies (e.g., β-hydroxybutyrate) or treating mice with SGLT2 inhibitors, can reduce protein hyperacetylation and improve diastolic function. Abbreviations: SIRT3 = sirtuin 3, ROS = reactive oxygen species, β-OH = β-Hydroxybutyrate, SGLT2i = sodium glucose transport protein 2 inhibitors.

The acetylation of mitochondrial proteins is crucial in the regulation of mitochondrial function. In HFpEF, protein hyperacetylation as a result of altered deacetylase activity or an increase in mitochondrial acetyl-CoA concentration contributes to the development and progression of heart failure (Figure 5) [221]. Alterations in mitochondrial protein acetylation have also been reported in diabetic cardiomyopathy [222] and hypertension [223], confirming a role for protein acetylation in diastolic dysfunction. Hyperacetylation has also been associated with inflammation, which exacerbates heart failure [224].

Sirtuin-3 is a mitochondria-specific NAD^+^-dependent deacetylase that plays a crucial role in mitochondrial homeostasis and disease [225]. Sirtuin-3 targets a plethora of mitochondrial proteins influencing energy production, oxidative stress, mitochondrial dynamics, and proteostasis [225]. In preclinical mouse models, sirtuin-3 knockout significantly elevated myocardial fibrosis, hypertrophy, and ROS formation [226,227]. Furthermore, inducing HFpEF in sirtuin-3 knockout mice with a three-hit model (high-fat diet coupled with deoxycorticosterone pivalate injections) exacerbated mitochondrial hyperacetylation in the heart, resulting in a more severe phenotype than the wildtype three-hit HFpEF mice [224]. A reduction in sirtuin-3 expression is evident in animal models of metabolic syndrome [228], HFpEF [6], and diabetic cardiomyopathy [229], signifying the importance of sirtuin-3 in disease. As sirtuin-3 is altered in disease and is mitochondrial-specific, novel therapeutics that increase sirtuin-3 activity are being investigated in the context of diastolic heart disease.

#### 6.2.1. Therapeutic Strategies Targeting Protein Acetylation

Honokiol is a compound derived from the bark of magnolia trees that increases sirtuin-3 levels [230]. As an activator of sirtuin-3, honokiol acts as a novel therapeutic that could reduce protein acetylation and ameliorate diastolic dysfunction. The reduction in sirtuin-3 reported in transaortic constricted mice is rescued by honokiol treatment [230]. Honokiol treatment also prevented the development of hypertrophy in transaortic constricted mice and reduced the development of fibrosis, demonstrating the efficacy of sirtuin-3 activation in the context of hypertrophy [230]. By increasing sirtuin-3 expression, honokiol reduced protein acetylation, which is proposed to reduce the activation of hypertrophic signaling pathways [230]. Furthermore, although evidence of honokiol efficacy in the treatment of diabetic cardiomyopathy has yet to be confirmed, honokiol has been reported to ameliorate the mitochondrial dysfunction associated with type II diabetic mouse models [222,231]. Hence, further work should investigate honokiol in the context of diastolic heart disease and test the efficacy of honokiol in the clinic.

Interestingly, the SGLT2 inhibitors canagliflozin, dapagliflozin and empagliflozin have been reported to increase sirtuin-3 expression in a mouse model of salt-induced cardiac hypertrophy [232]. Of the three SGLT2 inhibitors, canagliflozin had the most significant increase in sirtuin-3 expression [232]. Canagliflozin is proposed to stimulate sirtuin-3 expression by inhibiting p300-mediated histone acetylation [232]. The increase in sirtuin-3 expression may contribute to the efficacy of SGLT2 inhibitors in the treatment of HFpEF.

**Figure 6:**
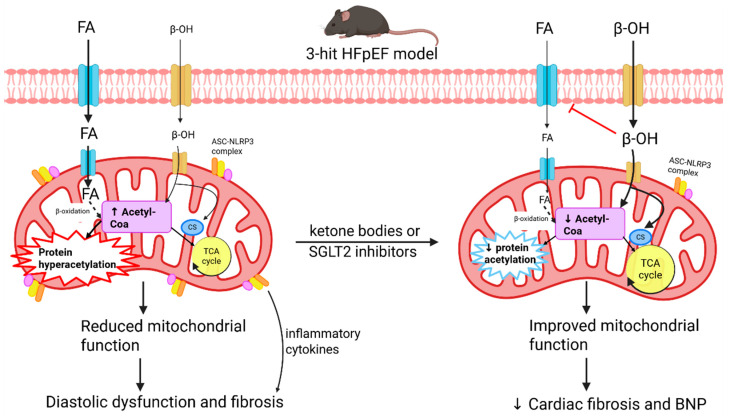
Ketone bodies and SGLT2 inhibitors can improve the HFpEF phenotype by increasing the ketone body β-hydroxybutyrate to reduce protein acetylation. The 3-hit HFpEF mouse model exhibits the characteristic fibrosis and diastolic dysfunction [224]. A reduction in ketone bodies in HFpEF contributes to an increase in the acetyl-CoA pool, which results in protein hyperacetylation. The combination of protein hyperacetylation and an increase in the ASC-NLRP3 complex which promotes an inflammatory state contributes to the diastolic dysfunction and fibrosis in HFpEF [224]. Treatment of the 3-hit HFpEF mice increases circulating β-hydroxybutyrate, inhibiting fatty acid uptake into the cell. Reduced fatty acid uptake results in a reduction in the acetyl-CoA pool, reducing protein acetylation. Improvements in mitochondrial function as a consequence of ketone body and SGLT2 inhibitor treatment reduced cardiac fibrosis and the cardiac marker BNP in the 3-hit mouse model [224]. The arrows show the movement of FA and β-OH into the cardiac myocyte and mitochondria and the resulting signaling cascades. Abbreviations: FA = fatty acid, β-OH = β-Hydroxybutyrate, ASC-NLRP3 = apoptosis-associated speck-like protein with caspase recruitment domain–NOD-like receptor protein 3, CS= citrate synthase, and BNP = brain natriuretic peptide.

Mitochondrial protein hyperacetylation has also been associated with inflammation, contributing to and exacerbating diastolic dysfunction (Figure 6) [224]. Hyperacetylation promotes the formation of the NOD-like receptor protein 3 (NLRP3) inflammasome and the apoptosis-associated speck-like protein, with the caspase recruitment domain (ASC) contributing to the pro-inflammatory phenotype evident in metabolic syndrome and HFpEF [224]. A reduction in ketone bodies such as β-hydroxybutyrate is also evident in HFpEF [224]. Deng Y. et al. reported that the treatment of the HFpEF three-hit mouse model with either ketone esters or the SGLT2 inhibitor empagliflozin increased circulating β-hydroxybutyrate, lowered protein acetylation, and ameliorated the HFpEF phenotype, as characterized through a reduction in brain natriuretic peptide, fibrosis, and an attenuation of hypertension and lung edema (Figure 6) [224]. The increase in β-hydroxybutyrate reduced ASC-NLRP3 formation and reduced pro-inflammatory cytokine-induced mitochondrial dysfunction and fibrosis [224]. The reduction in protein acetylation could occur both in wildtype and sirtuin-3 knockout mice, suggesting that β-hydroxybutyrate promotes deacetylation of proteins independently of sirtuin-3 [224]. Rather, β-hydroxybutyrate reduced the mitochondrial acetyl-CoA pool by suppressing fatty acid uptake and caspase recruitment (Figure 6) [224]. As ketone metabolism is altered in diastolic heart disease and linked to protein acetylation, targeting ketones such as β-hydroxybutyrate may improve diastolic dysfunction. It is important to note that although this study reports a reduction in end diastolic volume upon ketone ester or empagliflozin treatment, it did not report a difference in diastolic relaxation (−dt/dt) with either treatment compared to the vehicle control [224]. Hence, these data provide further evidence in support of the use of SGLT2 inhibitors in HFpEF, and further work should characterize the efficacy of ketones in treating diastolic dysfunction in both preclinical models and in the clinic.

#### 6.2.2. Clinical Evidence

SGLT2 inhibitors and honokiol are two therapeutic strategies that can increase protein acetylation in the heart. However, only SGLT2 inhibitors have clinical evidence supporting the clinical use of SGLT2 inhibitors in HFpEF.

The mechanisms driving the positive cardiac effects of SGLT2 inhibitors in HFpEF are currently being investigated. Cardiac cells do not express SGLT2, suggesting that the positive effects of SGLT2 inhibitors on cardiac function occur indirectly through alternative pathways. Preclinical studies suggest that the benefits of SGLT2 inhibition are due to a reduction in fibrosis and an improvement in mitochondrial function through sirtuin-3 or the acetyl-CoA pool [45,224]. To confirm whether the positive effect of SGLT2 inhibitors is in part due to protein acetylation, studies should investigate the effect of SGLT2 inhibitors on myocardial sirtuin-3, acetyl-CoA pool, and protein acetylation in myocardial samples from patients.

#### 6.2.3. Translational Considerations

Current metabolic therapies have demonstrated reduced efficacy in HFpEF. Although sirtuin-3 activators, such as honokiol, have yet to be investigated clinically in HFpEF, the failures of other metabolic-targeted therapeutics should be investigated to improve the translation of honokiol and other sirtuin 3 activators in the clinic. This includes investigating the efficacy of honokiol in robust, preclinical animal models for HFpEF. Investigations into how protein acetylation could potentially benefit diastolic dysfunction through extensive mechanistic studies will also enhance the translatability of honokiol into the clinic. The use of honokiol in a cocktail therapy approach, which uses a combination of therapeutics targeting metabolic and other mechanistic pathways in HFpEF, should also be considered to tackle the diverse etiology of HFpEF.

### 6.3. Altered Substrate Handling: NAD^+^/NADH

NAD^+^ is a co-enzyme for redox reactions which acts as an electron acceptor in catabolic reactions and a co-substrate for NAD^+^-dependent enzymes. NADH is the reduced form of NAD^+^, which is hydrolyzed by complex I in the electron transport chain, supplying electrons for oxidative phosphorylation to produce ATP. The ratio of NAD^+^/NADH reflects the balance of NADH production and consumption and is used as an indicator of mitochondrial function. A reduction in NAD^+^ in the heart and circulation has been reported in HFpEF animal models and in HFpEF patients. As fatty acid oxidation, redox reactions, mitochondrial electron transfer, and ATP production are NAD^+^-dependent processes, the alterations in NAD^+^ are proposed to contribute to the establishment and persistence of diastolic dysfunction.

#### 6.3.1. Therapeutic Strategies Targeting Altered Substrate Handling

As NAD^+^ cannot be readily taken up by cells, NAD^+^ is produced via synthesis and salvage pathways involving tryptophan, nicotinic acid, nicotinamide (NAM), and nicotinamide riboside (NR). Supplementation of NAD^+^ precursors for the treatment of various cardiac disorders is well documented within the literature. Furthermore, NAD^+^ precursor supplementation is an attractive therapeutic option, as it is orally administered and is easily accessible to patients. Dietary supplementation of either NR or NAM has been reported to ameliorate diastolic dysfunction and hypertrophy in ZSF-1 obese rats, two-hit HFpEF mice, *dahl*-salt-sensitive hypertensive rats, and aged mice in the absence of altered ejection fraction [4,5,6]. By increasing circulating and cardiac NAD^+^ with NR or NAM supplementation, mitochondrial energetics can be restored, and improvements in fatty acid oxidation are observed [4,5,6]. Furthermore, NAD^+^ activates sirtuins, and in ZSF-1 rats, NAM supplementation increased titin deacetylation, increasing passive stiffness [4]. It is important to note that the authors did not report a difference in titin acetylation between the control and ZSF-1 obese rats; the difference was only observed in ZSF-1 obese rats and ZSF-1 + NAM obese rats [4].

#### 6.3.2. Clinical Evidence

Increasing NAD^+^ through supplementation is an attractive therapeutic strategy that has been extensively investigated in human clinical trials. The oral administration route results in relatively good compliance, and studies have demonstrated a good safety profile for NR supplementation [233]. In the context of the heart, NR supplementation has been investigated in HFrEF but not HFpEF. A 12-week treatment with 1000 mg NR twice daily significantly increased circulating NAD^+^ in the blood and improved mitochondrial function in patient peripheral blood mononuclear cells. However, the increase in circulating NAD^+^ was coupled with a lack of improvements in cardiac parameters (ejection fraction, LV filling pressure (E’/e), LV end diastolic and systolic volumes and LV global longitudinal strain), functional capacity assessed as the distance achieved during a 6 min walk test, and quality of life [233]. To understand why cardiac function was not improved despite increased circulating NAD^+^, studies need to assess NAD^+^ levels in patient myocardium, which is an outcome in an ongoing HFrEF clinical trial (NCT0458004).

#### 6.3.3. Translational Considerations

Similarly to other metabolic/mitochondrial targeted drugs, NR supplementation was unable to improve cardiac function in the context of HFrEF. To pursue the translation of NR into the clinic for HFpEF, we need to understand why NR supplementation was ineffective in the HFrEF patient cohort.

The clinical trial for NR supplementation in HFrEF identified that there was interindividual variability in the NAD^+^ response [233]. The interindividual variability suggests differences in the individual’s response to NR supplementation. Understanding why patients have variable responses to NR in increasing circulating NAD^+^ should be investigated further to optimize the use of NR in the clinic.

NR supplementation increased circulating NAD^+^, but whether there was a corresponding increase in myocardial NAD^+^ was not assessed in this study. It is possible that the lack of cardiac improvements in the HFrEF patients was due to the inability to increase myocardial NAD^+^. Improving the translation of NR supplementation insights from the ongoing clinical trial (NCT0458004), which will look at myocardial NAD^+^, will be important. If the circulating NAD^+^ does not increase myocardial NAD^+^, studies will need to consider why NAD^+^ supplementation in HFrEF patients is unable to increase myocardial NAD^+^.

Another issue to address before moving into clinical trials for NR or NAM supplementation in the context of HFpEF is the discrepancies in results reported in the preclinical animal models. In ZSF-1 obese rats, NAM supplementation improved both the ATP/ADP ratio and phosphocreatine/ATP ratio in the left ventricle; however, NR supplementation did not improve the ratios in HFpEF mice [4,5]. Furthermore, although two separate studies investigated NR supplementation in the two-hit HFpEF mouse model, Koay et al. reported that a 4-week NR supplementation increased sirtuin-3 expression, while Tong et al. reported that an 8-week NR supplementation did not alter sirtuin-3 expression in the mice [4,5]. Standardizing the treatment type, dose, and the animal models used to test these therapies is required to validate the benefits of NR and NAM supplementation in HFpEF.

### 6.4. The Voltage-Dependent Anion Channel

The voltage-dependent anion channel (VDAC) is a β-barrel channel situated on the outer mitochondrial membrane [234,235]. Metabolic flux in and out of the mitochondria is predominantly regulated through VDAC, and molecular transport is regulated by the open and closed states of the channel [236,237,238,239]. In the open state, VDAC favors the transport of ADP, ATP, and small inorganic phosphates [234]. In the closed state, VDAC becomes impermeable to ADP and ATP and more permeable to cations including Ca^2+^ [234]. In cardiac myocytes, VDAC1 and VDAC2 are expressed [240]. VDAC2 is essential to cardiac function, as embryonic knockout of VDAC2 is embryonically lethal, and a cardiac-specific deletion of VDAC2 results in dilated cardiomyopathy, fibrosis, and alterations in calcium handling and action potentials [241,242]. Conversely, VDAC1 embryonic knockout mice are still viable despite the presence of mitochondrial dysfunction [243]. As VDAC is a crucial mitochondrial protein involved in metabolic flux, VDAC may be a potential therapeutic target to treat diastolic heart disease.

#### 6.4.1. VDAC in Diastolic Heart Disease

VDAC may be associated with the L-type calcium channel, suggesting a possible role of VDAC in modulating L-type calcium channel-dependent regulation of mitochondrial function in cardiac myocytes. In wildtype and *cTnI-G203S* hypertrophic mice, a pull-down proteomics assay identified that F-actin and VDAC immunoprecipitated with the L-type calcium channel [90]. Furthermore, application of a VDAC-binding peptide was able to hyperpolarize the mitochondrial membrane potential, as measured by an increase in JC-1 fluorescence, mimicking the increase in mitochondrial membrane potential measured after activation of the L-type calcium channel [90,91]. These data suggest that VDAC may act as a contact point between the cytoskeleton and the mitochondria. VDAC’s structural–functional relationship with the cytoskeleton provides an opportunity to target the interaction between VDAC and the cytoskeleton to disrupt the maladaptive mechanosensitive signaling between the ECM, L-type calcium channel, and the mitochondria. Altering the interaction between VDAC and the cytoskeleton (and consequently the L-type calcium channel) may ameliorate the hypermetabolic state in hypertrophic cells. However, this has yet to be investigated, and further work should characterize how VDAC mediates the communication between the L-type calcium channel and the mitochondria in hypertrophy.

#### 6.4.2. Translational Considerations

The important structural–functional role of VDAC in the heart acts as a novel therapeutic target for the treatment of diastolic heart disease. However, there is a lack of preclinical evidence for pharmacological agents that target the interaction between VDAC and the cytoskeleton to alleviate the hypermetabolic state present in diastolic heart diseases. Directly inhibiting VDAC function will be maladaptive due to VDAC’s crucial role in mitochondrial function. The development of pharmacological agents should be tested to ensure that the cytoskeletal uncoupling from VDAC does not impact VDAC’s important roles in regulating metabolic flux and mitochondrial calcium transport. Such considerations are important to prevent maladaptive effects, which may result in cardiac dilation and arrythmias.

## 7. Conclusions

Diastolic heart disease is a complex disease that results in cardiac hypertrophy, pathological ECM remodeling, and metabolic changes that contribute to the diastolic dysfunction. Despite the well-established functional relationship between the ECM, cytoskeleton, and mitochondria, it remains a challenge to develop effective treatments. Most therapeutics identified in preclinical studies do not progress to clinical trials due to a lack of improvement in diastolic functional parameters and/or due to adverse events. The efficacy of current therapeutics will be enhanced by refining preclinical animal models to better recapitulate the complex pathophysiology of HFpEF. Refinement of the preclinical models will provide opportunities to better characterize the mechanisms contributing to diastolic dysfunction. The use of combination therapies which target multiple mechanistic pathways, such as the ECM, cytoskeleton, and mitochondria, may also improve the efficacy of therapies and improve the clinical translation of these therapeutics into the clinic.

## Abbreviations

ADP	Adenine diphosphate
AHNAK	Neuroblast-associated differentiation protein/desmoyokin
AID	Alpha interaction domain
AID-TAT	Alpha interaction domain—transactivator of transcription
ASC	Apoptosis-associated speck-like protein with caspase recruitment domain
ATP	Adenine triphosphate
CaMKII	Ca^2+^/Calmodulin-dependent kinase II
cGMP	Cyclic guanosine monophosphate
ECM	Extracellular matrix
ERK	Extracellular signal-regulated kinase
GLP-1	Glucagon-like peptide-1
HDAC	Histone deacetylase
HFpEF	Heart failure with preserved ejection fraction
HFrEF	Heart failure with reduced ejection fraction
ICTP	Carboxy-terminal telopeptide of collagen type I
LVOT	Left ventricular outflow tract
MEF2	Myocyte enhancer factor 2
MMP	Metalloprotease
N2B-U	N2B unique sequence flanked by Ig domains
NAD+	Nicotinamide adenine dinucleotide
NADH	Nicotinamide adenine dinucleotide (reduced form)
NAM	Nicotinamide
NFAT	Nuclear Factor of Activated T-cells
NLRP3	NOD-like receptor protein
NOX	NADPH oxidase
NR	Nicotinamide riboside
PEVK	A structurally disordered region rich in proline, glutamate, valine, and lysine
p-FAK	Phosphorylated focal adhesion kinase
PICP	Carboxy-terminal propeptide of type I procollagen
PIIINP	Amino-terminal propeptide of type III procollagen
PINP	Amino-terminal propeptide of type I and III procollagen
PKA	Protein kinase A
PKC	Protein kinase C
PKG	Protein kinase G
RBM20	RNA-binding motif protein 20
ROS	Reactive oxygen species
SGLT2	Sodium glucose transporter 2
SORBS2	Sorbin and SH3 domain containing 2
TGF-β	Tissue growth factor beta
TIMP	Tissue inhibitors of metalloproteases
TRPM4	Transient receptor potential melastatin 4
VDAC	Voltage-dependent anion channel
ZSF-1	Zucker fatty and spontaneously hypertensive
